# Characterization of homologous sphingosine-1-phosphate lyase isoforms in the bacterial pathogen *Burkholderia pseudomallei*[S]

**DOI:** 10.1194/jlr.M071258

**Published:** 2016-12-29

**Authors:** Christopher J. McLean, Jon Marles-Wright, Rafael Custodio, Jonathan Lowther, Amanda J. Kennedy, Jacob Pollock, David J. Clarke, Alan R. Brown, Dominic J. Campopiano

**Affiliations:** EastChem School of Chemistry* University of Edinburgh, Edinburgh EH9 3FJ, United Kingdom; Institute of Quantitative Biology, Biochemistry, and Biotechnology, School of Biological Sciences,† University of Edinburgh, Edinburgh EH9 3FJ, United Kingdom; School of Biosciences,§ University of Exeter, Exeter EX4 4QD, United Kingdom

**Keywords:** sphingolipids, pyridoxal 5′-phosphate, assay, fatty aldehyde dehydrogenase

## Abstract

Sphingolipids (SLs) are ubiquitous elements in eukaryotic membranes and are also found in some bacterial and viral species. As well as playing an integral structural role, SLs also act as potent signaling molecules involved in numerous cellular pathways and have been linked to many human diseases. A central SL signaling molecule is sphingosine-1-phosphate (S1P), whose breakdown is catalyzed by S1P lyase (S1PL), a pyridoxal 5′-phosphate (PLP)-dependent enzyme that catalyzes the cleavage of S1P to (*2E*)-hexadecenal (2E-HEX) and phosphoethanolamine. Here, we show that the pathogenic bacterium, *Burkholderia pseudomallei* K96243, encodes two homologous proteins (S1PL2021 and S1PL2025) that display moderate sequence identity to known eukaryotic and prokaryotic S1PLs. Using an established MS-based methodology, we show that recombinant S1PL2021 is catalytically active. We also used recombinant human fatty aldehyde dehydrogenase to develop a spectrophotometric enzyme-coupled assay to detect 2E-HEX formation and measure the kinetic constants of the two *B. pseudomallei* S1PL isoforms. Furthermore, we determined the X-ray crystal structure of the PLP-bound form of S1PL2021 at 2.1 Å resolution revealing that the enzyme displays a conserved structural fold and active site architecture comparable with known S1PLs. The combined data suggest that *B. pseudomallei* has the potential to degrade host SLs in a S1PL-dependent manner.

Sphingolipids (SLs) are a diverse family of natural metabolites composed of a fatty acyl chain attached to a polar headgroup derived from L-serine that together form the sphingoid base core (1). Amphipathic in nature, SLs are important structural components of eukaryotic membranes that can associate with each other and with other membrane components (such as cholesterol and proteins) in regions called lipid rafts (2). As well as this structural role, SLs are now recognized as fundamental signaling molecules with a vast array of functions within cells. A “sphingodynamics” model has been proposed to encompass both intra- and extracellular signaling by all bioactive SL intermediates and their metabolites (3). Sphingosine-1-phosphate (S1P) is one such SL that regulates a plethora of physiological processes in humans by activating five different G protein-coupled S1P receptors (named S1PRs 1–5) (4). Perturbing the cellular levels of S1P can have serious consequences and has been linked to diseases such as cancer, diabetes, atherosclerosis, osteoporosis, and neurodegeneration (5, 6). The balance between S1P and ceramide, another signaling molecule that often has opposing cellular effects to S1P, is governed by a “sphingolipid rheostat” (6, 7). Remarkably, the pyridoxal 5′-phosphate (PLP)-dependent enzyme, S1P lyase (S1PL), is the only known enzyme capable of irreversibly removing sphingoid bases from the SL pool. S1PL catalyzes the retro-aldol-like breakdown of S1P to (*2E*)-hexadecenaldehyde (2E-HEX) and phosphoethanolamine (PE) (**Fig. 1**). In eukaryotes, the 2E-HEX is oxidized by fatty aldehyde dehydrogenase (FALDH; also known as ALDH3A2) to the hexadecenoic acid, which links the SL and glycerol phospholipid metabolic pathways (8). Saba, Hannun, and colleagues were the first to clone a S1PL gene (also known as *dpl1* and *BST1*) from *Saccharomyces cerevisiae* in 1997 (9) and, subsequently, the human homolog was characterized in 2000 (10). Because S1P plays a role in numerous diseases, enzymes in the S1P biosynthetic and degradative pathways are now seen as attractive drug targets (3). To that end, Novartis recently published results of a medicinal chemistry study that identified potent and specific inhibitors of human S1PL (11).

**Fig. 1.**
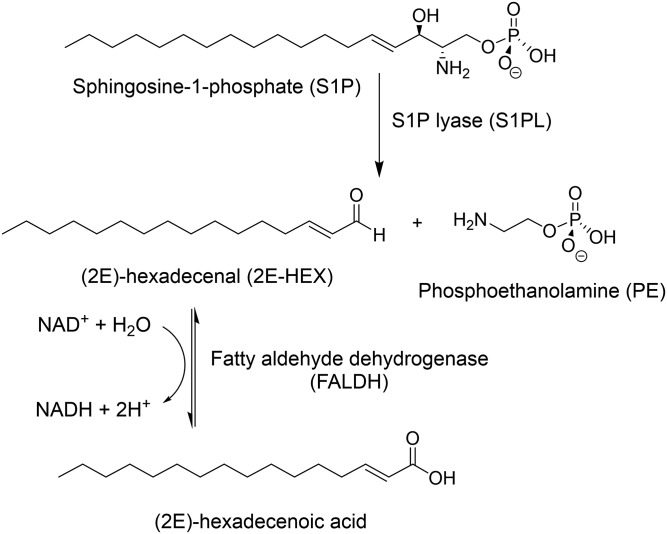
Metabolism of S1P. S1PL catalyzes the degradation of S1P to 2E-HEX and PE. The 2E-HEX product is a substrate for the NAD^+^-dependent enzyme, FALDH, which oxidizes 2E-HEX to the fatty acid with the concomitant reduction of the NAD^+^ cofactor.

Nearly all Gram-negative bacteria such as *Escherichia coli*, *Legionella pneumophila*, and *Burkholderia pseudomallei* contain lipopolysaccharides as the major lipid component in their cell membranes. In contrast, the number of bacteria that produce SLs, examples being *Sphingomonas spp* (12) and *Myxobacterium spp* (13), is relatively small. However, it is interesting to note that many of the bacteria that are part of the human microbiome, such as *Bacteroides fragilis* and *Porphyromonas gingivalis*, also produce SLs (14–16). This raises the interesting question of what is the function of bacterial SLs? Moreover, if such species contain an S1PL enzyme, what role does it play in bacterial metabolism in general and in the host-microbe interaction in particular? Recent studies have begun to address these questions. A bacterial S1PL homolog (LegS2) was identified in the intracellular human pathogen, *L. pneumophila* (17, 18), and its role as a virulence factor in the pathogenic mechanism of this organism was explored. *B. pseudomallei* is an intracellular Gram-negative pathogen that causes serious disease in humans and animals and, as such, is classified by the Centre for Disease Control as a category B biothreat agent. *B. pseudomallei* infection results in melioidosis, a febrile and potentially fatal disease that has a high mortality rate (19). To date, there have been no studies on SL metabolism in *Burkholderia*, so we began by investigating to determine whether this species contains any of the known SL metabolic enzymes, and the recent work on the *L. pneumophila* focused our attention on S1PL. We searched the *B. pseudomallei* K96243 genome (20, 21) and, surprisingly, found two open reading frames (ORFs), *BPSS2021* and *BPSS2025*, whose gene products (S1PL2021 and S1PL2025, respectively) displayed high sequence identity to each other and moderate sequence conservation to confirmed S1PLs from bacteria, yeast, and humans. We felt that this unusual gene duplication merited further study; thus, we set out to characterize the biochemical properties of these putative *B. pseudomallei* S1PLs as a prelude to investigating their role in the host-pathogen interaction.

A number of assays that monitor S1PL activity have been reported in the literature. Many use either radioactive (e.g., ^3^H or ^14^C) or fluorescently-labeled (e.g., BODIPY) S1P analogs that generate labeled 2E-HEX aldehyde that can be detected by scintillation counting or fluorescent methods, respectively (22–25). Other assays rely on the chemical derivatization of the HEX aldehyde product using chemical reagents, then applying MS to quantify the amount of material (LC-ESI-MS/MS, GC-MS, HPLC-ESI-QTOF, etc.) (26–28). Each approach has its merits with respect to sensitivity, limit of detection, and the ability to work with purified enzyme, cell-free extracts, or in whole cells. However, the drawbacks include the use of radioactive or nonnatural substrates, limited throughput, and the requirement for the extraction of the S1PL reaction products. First, using an established MS-based methodology, we validated that the recombinant S1PL2021 displayed S1PL activity (26). Then, to further characterize the two closely-related *B. pseudomallei* S1PLs, we generated a coupled spectrophotometric method for the convenient measurement of S1PL activity. This assay couples the activity of these new S1PLs with a recombinant form of human FALDH that is part of the 2E-HEX catabolic pathway. In this way, we were able to measure the production of 2E-HEX via the reduction of the FALDH NAD^+^ cofactor to NADH. Furthermore, we determined the X-ray crystal structure of the S1PL2021 isoform at 2.1 Å and revealed that it has features in common with bacterial, yeast, and human S1PL homologs. As well as reporting a convenient assay for S1PL activity, this study lays the foundation for future studies of the role of these novel S1PLs in the growth and pathogenicity of *Burkholderia*.

## MATERIALS AND METHODS

### Materials

Plasmids and *E. coli* competent cells for cloning were purchased from Novagen and New England BioLabs. The *E. coli* BL21 (DE3) competent cells used for protein expression were from Agilent. All chromatography columns were from GE Healthcare. Hotstart *Pfu* polymerase was from Agilent. Restriction enzymes were from New England BioLabs. Oligonucleotide primers were sourced from Sigma. Substrate S1P (CAS number: 26993-30-6) was from Cayman Chemical Co. 2E-HEX was synthesized in house using a published method (29). All other buffers and reagents were sourced from Sigma.

### Sequence alignment and prediction tools

Sequence data searches were performed using the BLAST suite of programs. Multiple sequence alignments were carried out using the Clustal series of programs (30) with structural modeling performed using the Phyre2 server (31). To find S1PL homologs in the curated *B. pseudomallei* genome (www.burkholderia.com), the S1PL sequence from *Symbiobacterium thermophilim* [UniProt code, Q67PY4; Protein Data Bank (PDB) code, 3MAU] was used to carry out a standard BLAST analysis. This recovered two hit genes, annotated as *BPSS2021* (UniProt code, Q63IP8) and *BPSS2025* (Uniprot code, Q63IP4) with moderate sequence identities to the template (43% and 44%, respectively). The encoded proteins, referred to as S1PL2021 and S1PL2025, share ∼86% sequence identity.

### Cloning and expression of *BPSS2021* (S1PL2021)

Sequence analysis suggested that the *BPSS2021* gene could be expressed from two start codons to give two ORFs. Therefore, PCR forward and reverse primers were designed based on the longest ORF (S1PL2021 N-EXT) using the following primers: S1PL2021 forward, 5′ ***CCATGG***TGCGTTCGATCGC 3′ S1PL2021 reverse, 5′ ***CTCGAG***CGGACAGTCGGTAAACAG 3′. These primers incorporated *Nco*I and *Xho*I restriction sites giving a C-terminal six-histidine-tagged fusion. A positive pGEM/S1PL2021 N-EXT clone was digested and the isolated S1PL gene ligated into pET-28a. This gave a pET-28a/S1PL2021 N-EXT construct that expressed a protein of 500 amino acids. To clone the shorter ORF (S1PL2021), a forward primer was designed to initiate expression from an ***ATG*** start codon 60 base pairs downstream of the annotated start codon: S1PL2021 TRUNC forward, 5′ ***CCATGG***ATCTCGAAGAAGG 3′. This PCR product was also cloned into pET-28a via a pGEM intermediate to give a pET-28a/S1PL2021 plasmid encoding a protein of 480 amino acids. Plasmids pET-28a/S1PL2021 N-EXT and pET-28a/S1PL2021 were used to transform *E. coli* BL21 (DE3) competent cells and selection was carried out on agar plates containing kanamycin (30 μg/ml; LB/Kan^30^). Single colonies were used to inoculate 2 × 250 ml of LB/Kan^30^ broth and overnight cultures were grown at 37°C with shaking at 250 rpm. The overnight cultures were used to inoculate 4 l of fresh LB/Kan^30^ broth and grown to an A_600_ of 0.6–0.8. Protein expression was induced by addition of IPTG to a final concentration of 0.1 mM and growth was continued for 5 h at 30°C. Cells were harvested by centrifugation (Sorvall RC5-B centrifuge) at 11,385 *g* for 15 min at 4°C.

### Cloning and expression of the S1PL2021 K271A mutant

S1PL2021C mutants were prepared using the method of Liu and Naismith (32) with primers that were designed to introduce the relevant mutations: K271A forward, 5′ CGATACGCAC***GCG***TTCGGCTACGGCCCGAAGGGC 3′ K271A reverse, 5′CGCCCTTCGGGCCGTAGCCGAA***CGC***GTGCGTATCG 3′. PCR reactions were set up using Hotstart *Pfu* polymerase with completed reactions digested with *Dpn*1 enzyme to degrade template DNA. The post digestion mixture was transformed into high cloning efficiency cells (C2987; New England BioLabs) and spread on LB/Kan^30^ plates. Colonies were picked into LB/Kan^30^ and grown overnight with plasmid DNA extracted from cells using a Qiaprep miniprep kit (Qiagen). Sequencing was performed to ensure that mutagenesis had occurred. Expression of the S1PL2021 mutants mirrored that of the wild-type with the exception that induction temperature was reduced to 28°C.

### Cloning and expression of *BPSS2025* (S1PL2025)

The coding sequence for the *BPSS2025* gene was amplified from *B. pseudomallei* K96243 chromosomal DNA using primers S1PL2025 forward, 5′ ***CCATGG***ATCTGGAGGAAGGCATCAG 3′ and S1PL2025 reverse, 5′ ***CTCGAG***AAGCGGGCAATCCGTGAAC 3′ to give an expressed protein sequence that aligned with the shorter S1PL2021 form described above. The purified PCR product was cloned into the pGEM T-Easy vector by TA ligation and positive clones were isolated and sequenced to confirm the identity of the gene. A positive pGEM/S1PL2025 clone was digested with *Nco*I and *Xho*I, and the isolated S1PL gene ligated into pET-28a to give a C-terminally six His-tag protein of 480 amino acids. Expression of the gene and purification of the protein product were essentially identical to that for S1PL2021.

### Cloning and expression of full-length and truncated FALDH

The full-length FALDH gene sequence (coding isoform 2) was purchased as a cDNA clone from Imagenes in a pOTB7 vector (UniProt code, P51648). Full-length FALDH (isoforms 1 and 2) is predicted to contain a C-terminal membrane-bound helix, thus using this as a template. Full-length and truncated versions for the FALDH isoform 1 (the major forward and reverse primers with *Nde*I/*Nco*I and *Xho*I restriction enzyme sites: FALDH forward, 5′ ***CATATG***GAGCTCGAAGTCCGGCGGGTCCGACAGG; FALDH reverse, 5′ ***CTCGAG***(TCA)TAATATTCCGCCTTGACAAGCACAGCGGCTACAAT; Truncated FALDH reverse, 5′ ***CTCGAG***ATCCACCTTTGACTGGCTGTTGGG. The purified PCR products were cloned into pGEM, sequence verified, and then cloned into pET-28a as described for the S1PL constructs as an N-terminally hexa-his-tagged protein. pET-28a plasmids containing both versions of FALDH (full-length and C-terminal truncation) were used to transform *E. coli* BL21 (DE3) competent cells and selection was carried out on LB/Kan^30^ plates. Single colonies were used to inoculate 2 × 250 ml of LB/Kan^30^ broth and overnight cultures were grown at 37°C with shaking at 250 rpm. The overnight cultures were used to inoculate 4.5 l of fresh LB/Kan^30^ broth and grown to an A_600_ of 0.6–0.8. Protein expression was induced by addition of IPTG to a final concentration of 0.1 mM and growth was continued for 5 h at 22°C. Cells were harvested by centrifugation (Sorvall RC5-B centrifuge) at 5,000 rpm for 15 min at 4°C.

### General procedure for the purification of six his-tagged enzymes (S1PL2021, S1PL2025, and FALDH)

All purification steps were carried out at 4°C. All buffers contained 20 mM HEPES (pH 7.5), 400 mM NaCl, and 10% glycerol. PLP (100 μM) was included in the buffer for S1PL isolation. Binding buffers also contained 10 mM imidazole and one EDTA-free protease inhibitor tablet (Roche). Wash buffer contained 30 mM imidazole with elution buffer containing 300 mM imidazole. Cell pellets (∼4 g) were resuspended in Ni-NTA binding buffer 10:1 (v/w). Cells were lysed by sonication (Soniprep 150) for 15 cycles (30s on, 30 s off) on ice. In the case of full-length FALDH, 0.1% N-lauroylsarcosine was added to aid solubilization of the recombinant protein. The lysed cell suspension was centrifuged at 20,000 rpm for 30 min. The cell-free extract was filtered (0.45 μm syringe filter) before addition of 2 ml Ni-NTA resin (Qiagen) pre-equilibrated with binding buffer. Binding was carried out by rotating the suspension at 4°C for 1 h. The resin was collected by gravity flow in a fritted plastic disposable column, washed with 30 ml wash buffer, and the bound protein eluted with 5 ml elution buffer. Fractions were analyzed by SDS-PAGE and the 300 mM imidazole eluate loaded onto a pre-equilibrated HiPrep™ 16/600 Superdex™ S-200 size exclusion column. Recombinant protein was eluted at a flow rate of 1 ml/min in buffer. The molecular weights of S1PL or FALDH were calculated from a calibration curve using known molecular mass standards (GE Healthcare). The purity of the recombinant proteins was analyzed by SDS-PAGE. The fractions were pooled and protein concentration was calculated by A_280_ using the relevant extinction coefficients as determined using the Scripps Protein Calculator.

### S1PL UV-visible spectroscopy

All UV-visible (UV-vis) spectra were recorded on a Cary 50 spectrophotometer (Varian) and analyzed using Cary WinUV software (Varian). S1PL enzymes (15–20 μM) were maintained in holo-form by dialyzing against buffer containing 20 mM HEPES (pH 7.5), 400 mM NaCl, 10% glycerol, and 100 μM PLP. Prior to spectroscopic measurements, excess PLP was removed using a PD-10 desalting column (GE Healthcare). A stock solution of S1P (1 mg/ml) was prepared in 1% Triton X-100 and sonicated. A stock solution of PE (100 mM) was prepared in water. S1P and PE were added to the enzymes and scans recorded between 300 and 500 nm at appropriate time intervals.

### Assay of S1PL activity by LC-MS

The activity of S1PL was determined by detecting the formation of the 2E-HEX semicarbazone (2E-HEX SC) product by LC-MS using the method of Berdyshev et al. (26). Briefly, an enzyme stock solution was prepared by dialyzing 10 μM S1PL in 20 mM HEPES (pH 7.5), 400 mM NaCl, and 100 μM PLP. A 2.6 mM S1P stock was prepared with additional sonication. Using these stocks, an enzyme assay was prepared to a final volume of 500 μl containing 6 μM S1PL and 0.26 mM S1P substrate in a reaction buffer containing 20 mM HEPES, 400 mM NaCl, and 0.1 mM PLP. Control assays were prepared accordingly and all samples were incubated for 20 min at 37°C with methanol then added to stop the reaction. The 2E-HEX was extracted and the semicarbazone derivative produced by heating reaction aliquots with 0.2 ml of 5 mM semicarbazide hydrochloride in methanol containing 5% formic acid at 40°C for 2 h. The semicarbazones were analyzed by MS with samples injected via an Agilent 1200 HPLC with interfaced autosampler (Agilent Technologies). Analysis was in ESI-positive mode on a micro-TOF2 (Bruker Daltonics) using a standard ESI source. Ion source conditions were as follows: the ESI voltage was held at 4.5 kV at a temperature of 200°C with a hexapole RF of 70 V peak to peak and skimmer 2 potential of 20 V.

### Assay of FALDH activity using UV-vis spectroscopy

The soluble version of FALDH, lacking its C terminus, was assayed to determine its suitability as a coupling enzyme with S1PL. This was done by monitoring the formation of NADH in the presence of a synthetic 2E-HEX substrate. Reaction aliquots were prepared to a final volume of 500 μl containing 1 mM NAD^+^, 500 nM FALDH in a reaction buffer containing 20 mM HEPES (pH 7.5) and 400 mM NaCl. Reactions were monitored by UV-vis at 340 nm for 30 min at 37°C. Subsequent assays were performed in a Synergy HT plate reader and the data plotted using GraphPad (nonlinear regression analysis) to gain kinetic data for FALDH. The reaction was linear over ∼5 min and the lower limit of detection was ∼4 μM 2E-HEX.

### S1PL:FALDH coupled-enzyme assay

S1PL enzyme assays were prepared to a final volume of 500 μl containing 6 μM S1PL, 1 mM NAD^+^, 500 nM FALDH in a reaction buffer containing 20 mM HEPES (pH 7.5), 400 mM NaCl, and 0.1 mM PLP. The reaction was initiated by the addition of S1P from the stock solution to give a final concentration of 400 μM. Control samples lacking enzyme or substrate were also prepared. Reactions were monitored by UV-vis at 340 nm for 30 min at 37°C on a spectrophotometer. For kinetic data, the assays were then transferred to a Synergy HT plate reader and these contained 6 μM S1PL, 1 mM NAD^+^, 500 nM FALDH in a reaction buffer containing 20 mM HEPES (pH 7.5), 400 mM NaCl, and 0.1 mM PLP (final well volume 150 μl). This time, substrate S1P (0–175 μM) was prepared in 1% Triton X-100 and added to separate reaction aliquots in triplicate. Reactions were monitored at 340 nm for 30 min at 37°C. Kinetic data were fit using GraphPad as described previously.

### X-ray crystallography

S1PL2021, at 6 mg/ml, was crystallized by hanging-drop vapor diffusion at 18°C. Crystallization experiments were set up in 96-well MRC plates with commercially available screens from Molecular Dimensions Limited with 100 nl drops of protein and 100 nl well solution and equilibrated against 70 μl of well solution. Crystals grew in a well solution of 20% (v/v) polypropylene glycol 400 and 10% (v/v) 1-propanol (Molecular Dimensions MIDAS screen). Crystals were cryoprotected with mineral oil and then flash-cooled by immersion in liquid nitrogen. Datasets were collected on beamline I03 at the Diamond Light Source (Didcot, UK) at 100 K using a Pilatus 6M detector. Diffraction data were integrated and scaled using XDS (33) and symmetry-related reflections were merged with Aimless (34). The resolution cut off used for structure determination and refinement was determined based on the CC1/2 criterion proposed by Karplus and Diederichs (35). The structure of S1PL2021 was determined by molecular replacement using a model based on PDB code 3MAD, modified to match the target sequence using Chainsaw (36). A single solution comprising two molecules in the asymmetric unit was found using Phaser (37). The initial model was rebuilt using Phenix.autobuild (38) followed by cycles of refinement with Phenix.refine (39) in the Phenix program suite (40) and manual rebuilding in Coot (41) The final model was refined with isotropic B-factors and the model was validated using MolProbity (42) Structural superimpositions were calculated using Coot. Crystallographic figures were generated with PyMOL (43)

## RESULTS

### Identification of putative *Burkholderia* S1PLs by BLASTp search

BLASTp hits for novel bacterial S1PLs were identified in the genomes of various species of *Burkholderia* by using the 507 amino acid sequence of the confirmed bacterial S1PL from *S. thermophilum*, *St*S1PL (UniProt code, Q67PY4), as the query sequence (20, 44). Several putative S1PLs with >40% sequence identity were identified in the known human pathogen, *B. pseudomallei*, and in *B. thailandensis*, a nonpathogenic model organism (45). Strikingly, all representatives of these species each encode two putative S1PLs, with the two genes located in close proximity within each species. This is exemplified by *B. pseudomallei* strain K96243, which contains two genes in close proximity with locus IDs of *BPSS2021* (S1PL2021) and *BPSS2025* (S1PL2025) that display 86.3% amino acid sequence identity between them and 43.9% and 43.1% identity, respectively, to the confirmed *St*S1PL (supplemental Figs. S1, S2). These two predicted S1PLs also contained the conserved residues involved in binding the PLP cofactor (44).

To characterize the recombinant forms of these putative S1PLs, we began by expressing S1PL2021 in *E. coli*, but the ORF displayed two potential start codons. The sequence identity between S1PL2021 with *St*S1PL was low at the N termini (supplemental Fig. S2), with the pairwise alignment beginning at M47 of *St*S1PL. This aligns well with a conserved MDLEEG motif found in S1PL2021 and the other S1PL homologs in *Burkholderia* species and two clones were prepared; one that began at the conserved MDLEEG motif (named S1PL2021) and another that expressed an extended construct [named S1PL2021 (N-EXT)].

### Purification and characterization of S1PL2021 and S1PL2021 N-EXT

Recombinant S1PL2021 was expressed in *E. coli* from plasmid pET-28a as a hexa-histidine (6His) C-terminal fusion. A standard purification procedure using immobilized nickel affinity chromatography and size-exclusion chromatography yielded milligram amounts of pure recombinant protein S1PL2021 that was predominantly dimeric in solution (supplemental Fig. S3A, B). A similar strategy was used to prepare recombinant S1PL2025 (supplemental Fig. S3C, D). Analysis of S1PL2021 suggested that PLP was bound to the protein because the UV-vis spectrum displayed absorbances at ∼330 and ∼420 nm that were characteristic of PLP-dependent enzymes (supplemental Fig. S3E). A similar PLP binding spectrum was observed with S1PL2025 (supplemental Fig. S3F). In contrast, the alternative construct, S1PL2021 N-EXT, purified in an identical manner to the S1PL2021, but failed to display any PLP binding (supplemental Fig. S3G). These results confirmed that these truncated versions of S1PL2021 and S1PL2025 bound PLP and these forms of the protein were used throughout the study.

### Substrate and product binding to S1PL2021

**Fig. 2.**
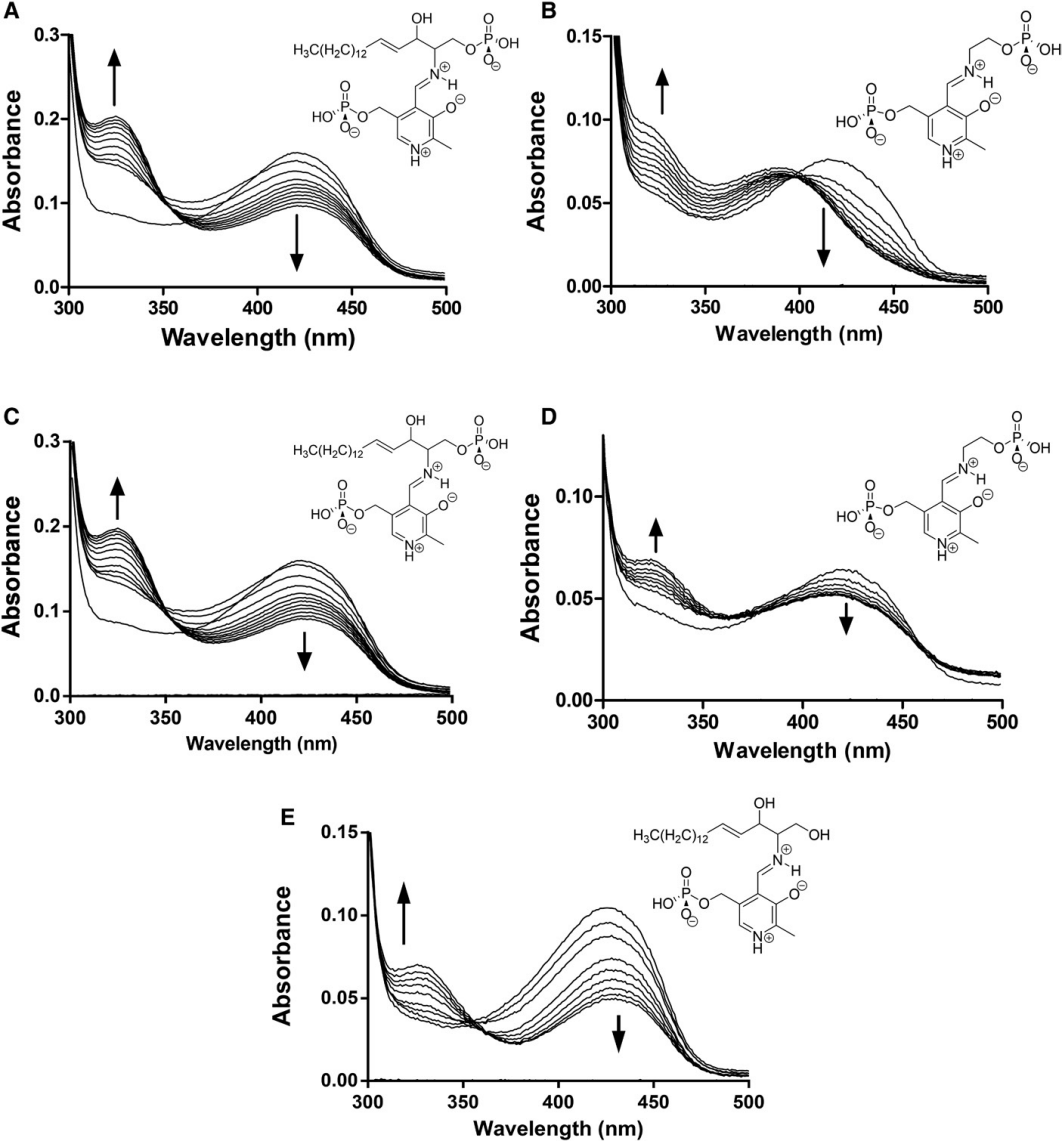
UV-vis spectroscopy analysis of purified S1PL2021. A: The UV-vis spectrum of PLP-bound S1PL2021 changes upon incubation with 100 μM S1P substrate in a time-dependent manner. Over 30 min (scanning every 3 mins), a decrease in absorbance at 420 nm, coupled to an increase in the 330 nm region is observed. The structure of the PLP-S1P external aldimine substrate is shown. B: Addition of 10 mM PE to S1PL2021 results in changes to the PLP spectrum over a longer timeframe (3 h, scanning every 30 min). There is a shift of the 420 nm peak to 395 nm and a broader increase in the 320–380 nm region associated with the formation of PLP-PE external aldimine (structure shown). C: Addition of 100 μM S1P to S1PL2025 leads to changes in the PLP UV-vis spectrum region similar to that observed with S1PL2021 (30 min, with scans every 3 min). D: Addition of 10 mM PE to S1PL2025 results in changes to the PLP spectrum over a longer timeframe (3 h, scanning every 30 min). E: Addition of 300 μM D-*erythro* sphingosine to S1PL2021 results in changes to the PLP spectrum (4 h, scanning every 30 min).

When the substrate S1P (100 μM) was added to the PLP-bound form of S1PL2021 (15 μM) and the resulting mixture analyzed by UV-vis spectroscopy, the peak at 420 nm decreased along with an increase in the 330 nm region over a period of 30 min, suggesting that S1P binds to S1PL2021 (**Fig. 2A**). Addition of the product PE (10 mM) to the protein also showed changes in the UV-vis region (maxima at ∼320 nm) with an additional blue shift to ∼395 nm, although this occurred at a much slower rate than for S1P (Fig. 2B). According to the putative S1PL mechanism proposed by Bourquin et al. (44), these spectroscopic changes are due to the formation of the PLP-S1P and PLP-PE external aldimine intermediates that are bound to S1PL (supplemental Fig. S4). We carried out similar UV-vis analysis of the binding of S1P and PE to the S1PL2025 enzyme (Fig. 2C, D). The changes in the UV-vis spectra were similar when we compared the incubation of S1PL2021 and S1PL2025 with S1P. In contrast, when we compared the spectra from the incubations of the product PE with both enzymes, we did not observe the 420–395 nm shift in the S1PL2025 enzyme and the changes took longer to occur compared with the S1PL2021 enzyme. These combined data suggest that, despite having highly similar amino acid sequences (86%), the two S1PL isoforms display small differences in their cofactor, substrate, and product binding that deserve further investigation. We also used UV-vis spectroscopy to show that S1PL2021 could also bind the nonphosphorylated substrate, D-*erythro* sphingosine (Fig. 2E). Addition of this substrate led to similar changes in the PLP region of the UV-vis spectrum that were observed for S1P, although this took significantly longer (∼4 h).

### Confirmation that S1PL2021 is an S1PL

**Fig. 3.**
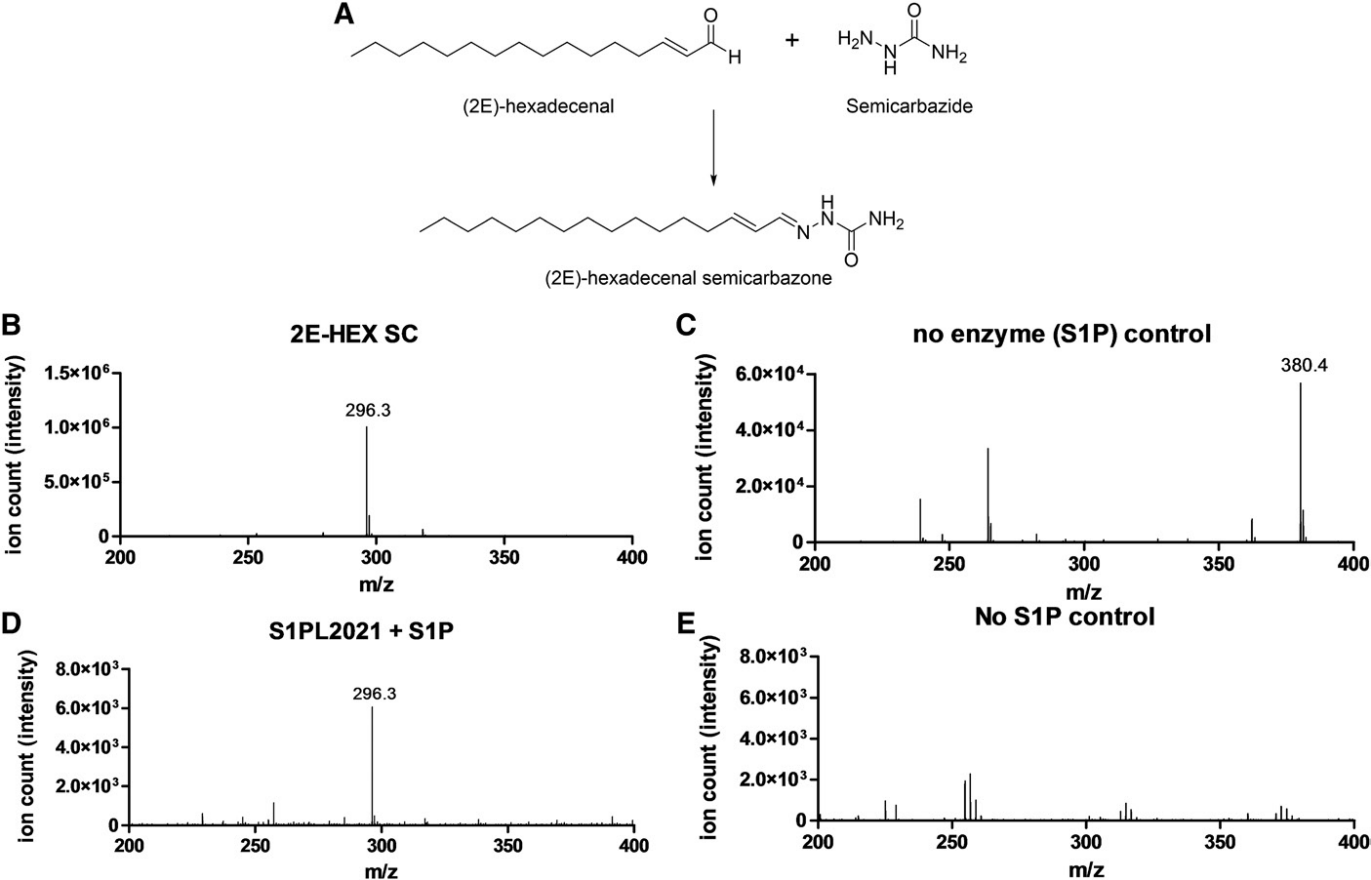
Analysis of S1PL2021 activity by the Berdyshev et al. (26) method. A: The reaction between 2E-HEX and semicarbazide leads to formation of the semicarbazone derivative (2E-HEX SC), which under ESI-MS gives an ion with a predicted value of *m/z* 296.3 Da ([M+H]^+^, C_17_H_34_N_3_O^+^). B: ESI-MS analysis of the 2E-HEX positive control reaction showing an ion with *m/z* 296.3 Da corresponding to the 2E-HEX SC. C: Incubation of S1P in buffer followed by semicarbazide derivitization in the absence of enzyme. The ion with *m/z* 296.3 Da is absent, but an ion with *m/z* 380.4 Da corresponding to S1P is observed ([M+H]^+^, C_18_H_38_NO_5_P = 380.2 Da, predicted). D: The S1PL2021 enzymatic reaction showing the formation of the 2E-HEX SC ion with *m/z* 296.3 Da confirming the activity of S1PL2021. E: The no substrate control lacking S1P and the 2E-HEX SC.

To determine whether the *B. pseudomallei* S1PL2021 and S1PL2025 displayed S1PL activity, we used an established MS-based method developed by Berdyshev et al. (26) that has been used to detect S1PL activity in mammalian liver microsomes. This method relies on extracting the 2E-HEX aldehyde product of the S1PL reaction and forming a stable semicarbazone derivative (2E-HEX SC, [M+H]^+^ C_17_H_34_N_3_O^+^, predicted *m*/*z* 296.3 Da, observed *m*/*z* 296.3 Da) using semicarbazide hydrochloride (**Fig. 3A**). Recombinant S1PL2021 was incubated with 260 μM S1P and aliquots taken for semicarbazone derivitization. Additional positive and negative controls were prepared and treated in an identical manner and the results analyzed using ESI-MS (Fig. 3B-E). The positive control used previously synthesized 2E-HEX and gave a single peak at *m*/*z* 296.3 Da matching the expected semicarbazone derivative (Fig. 3B). This peak was absent in the no enzyme control with the S1P substrate ([M+H]^+^, predicted C_18_H_39_NO_5_P^+^ *m/z* 380.2 Da, observed *m/z* 380.4 Da; Fig. 3C). The reaction catalyzed by S1PL2021 resulted in a signal with *m/z* 296.3 Da, indicating the production of the 2E-HEX SC species and confirming that S1PL2021 displayed S1PL activity (Fig. 3D). In contrast, the no substrate control lacked both of the above peaks (Fig. 3E).

### FALDH coupled assay suitability/optimization

**Fig. 4.**
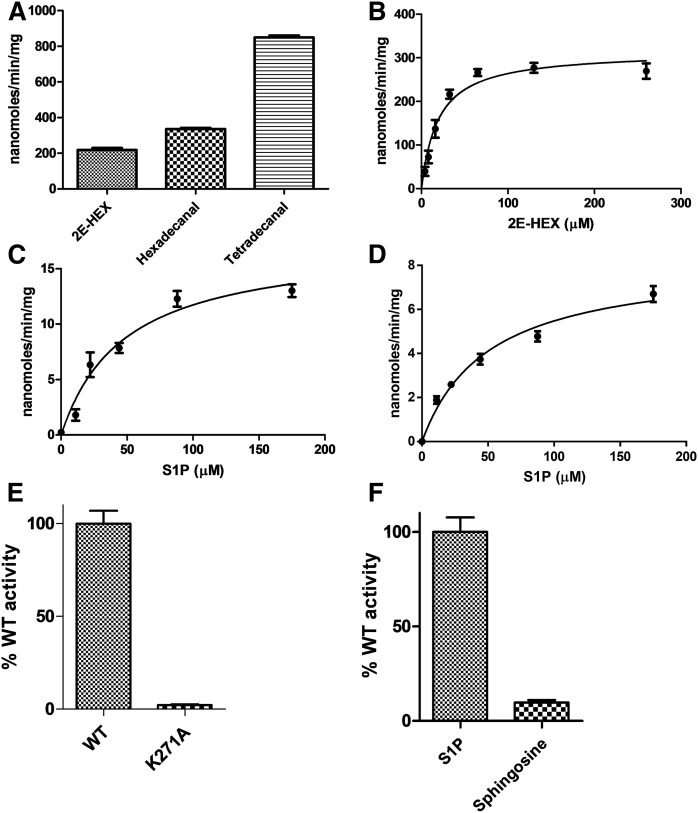
Kinetic analysis of FALDH and S1PL2021/S1PL2025. A: Recombinant human FALDH (0.5 μM) displays enzyme activity with aldehyde substrates, 2E-HEX, hexadecanal, and tetradecanal. B: Michaelis-Menten kinetic analysis of FALDH using 2E-HEX as a substrate (0–260 μM). The *K_M_* for 2E-HEX = 19.4 ± 3.2 μM and *k*_cat_ = 0.22 ± 0.01 s^−1^. C, D: Michaelis-Menten kinetic analysis using the S1PL/FALDH coupled assays with S1P as substrate (6.0 μM S1PL, 0.5 μM FALDH). The *K_M_* and *k*_cat_ values for S1P are: 48.2 ± 9.4 μM and 0.015 ± 0.003 s^−1^ for (C) S1PL2021 and 50.2 ± 8.3 μM and 0.008 ± 0.0005 s^−1^ for (D) S1PL2025. E: The S1PL2021 K271A mutant displays negligible activity when using the coupled assay. F: Use of the coupled assay to allow comparison of the S1PL20121 lyase activity with S1P (100 μM) and D-*erythro* sphingosine (300 μM) substrates.

The ESI-MS method confirmed that S1PL2021 displays S1PL activity; therefore, we went on to develop a convenient continuous spectroscopic assay in order to perform steady-state kinetic analysis of S1PL. We took advantage of the activity of FALDH, the next enzyme in the SL metabolic pathway (Fig. 1). The human FALDH (also known as ALDH3A2) has been cloned and recombinant enzyme has been isolated from *E. coli* by Lloyd et al. (46), so this presented an attractive candidate for the development of a coupled assay. A commercially available cDNA clone (Source BioScience, product code IRAUp969B0810D in plasmid pOTB7) was used to construct an expression clone of the full-length FALDH protein (isoform 1); however, in our hands, this initial construct was insoluble (data not shown). A sequence alignment between human FALDH and rat ALDH3A1, which lacks a hydrophobic C-terminal transmembrane (TM) region, guided the preparation of a second truncated clone (supplemental Fig. S5). This shorter variant expressed well in *E. coli* and we were able to isolate milligram quantities of recombinant His-tagged human FALDH (supplemental Fig. S6). By monitoring aldehyde substrate-dependent NADH formation at 340 nm, we established that the fully saturated substrates, hexadecanal and tetradecanal, and, most importantly, the unsaturated 2E-HEX, were substrates for this recombinant FALDH (**Fig. 4A**). Using varying 2E-HEX substrate concentrations (0–260 μM), the reaction was linear over the first 3 min and the limit of detection was determined to be ∼4 μM 2E-HEX (supplemental Fig. S7A). We performed Michaelis-Menten analysis and determined the kinetic values for 2E-HEX with *K_M_* = 19.4 ± 3.2 μM and *k*_cat_ = 0.22 ± 0.01 s^−1^ recorded (Fig. 4B, **Table 1**). The active FALDH was then coupled to both S1PL2021 and S1PL2025 in the presence of S1P and NAD^+^ at 30°C to determine the kinetic constants of both S1PL enzymes. The initial reaction rates were linear over a S1P concentration range of 0–175 μM. Therefore, S1PL activity could be quantified in a continuous fashion (down to a S1P substrate concentration of ∼10 μM) (supplemental Fig. S7B, C) and allowed the determination of the kinetic constants of both *B. pseudomallei* S1PLs. The values for each are as follows: S1PL2021 (*K_M_* = 48.2 ± 9.4 μM and *k*_cat_ = 0.015 ± 0.003 s^−1^) and S1PL2025 (*K_M_* = 50.2 ± 8.3 μM and *k*_cat_ = 0.008 ± 0.0005 s^−1^) (Fig. 4C, D; Table 1). Thus, this new assay offered a convenient way of measuring the activity of recombinant S1PL. Based on the S1PL sequence alignment, residue K271 of S1PL2021 is predicted to bind the essential PLP cofactor (supplemental Fig. S2). A S1PL2021 K271A mutant was prepared and, when used in the coupled assay, this variant was essentially inactive in comparison to the wild-type enzyme (Fig. 4E). We also used the assay to determine whether S1PL2021 used D-*erythro* sphingosine as a substrate and found that it displayed ∼8% activity compared with S1P (Fig. 4F).

**TABLE 1.** Michaelis-Menten kinetic data for recombinant human FALDH using 2E-HEX as substrate and the *B. pseudomallei* S1PL2021 and S1PL2025 enzymes using S1P as a substrate

	FALDH (2E-HEX as Substrate)	S1PL2021 (S1P as Substrate)	S1PL2025 (S1P as Substrate)
*V*_max_ (nmol/min/mg)	314.9 ± 13.9	17.5 ± 1.3	8.2 ± 1.8
*K_M_* (μM)	19.4 ± 3.2	48.2 ± 9.4	50.19 ± 8.3
*k*_cat_ (s^−1^)	0.22 ± 0.01	0.015 ± 0.003	0.008 ± 0.0005
*k*_cat_/*K_M_* (M^−1^s^−1^)	1.13 × 10^4^	3.2 × 10^2^	1.5 × 10^2^

### Structural characterization of S1PL2021

**Fig. 5.**
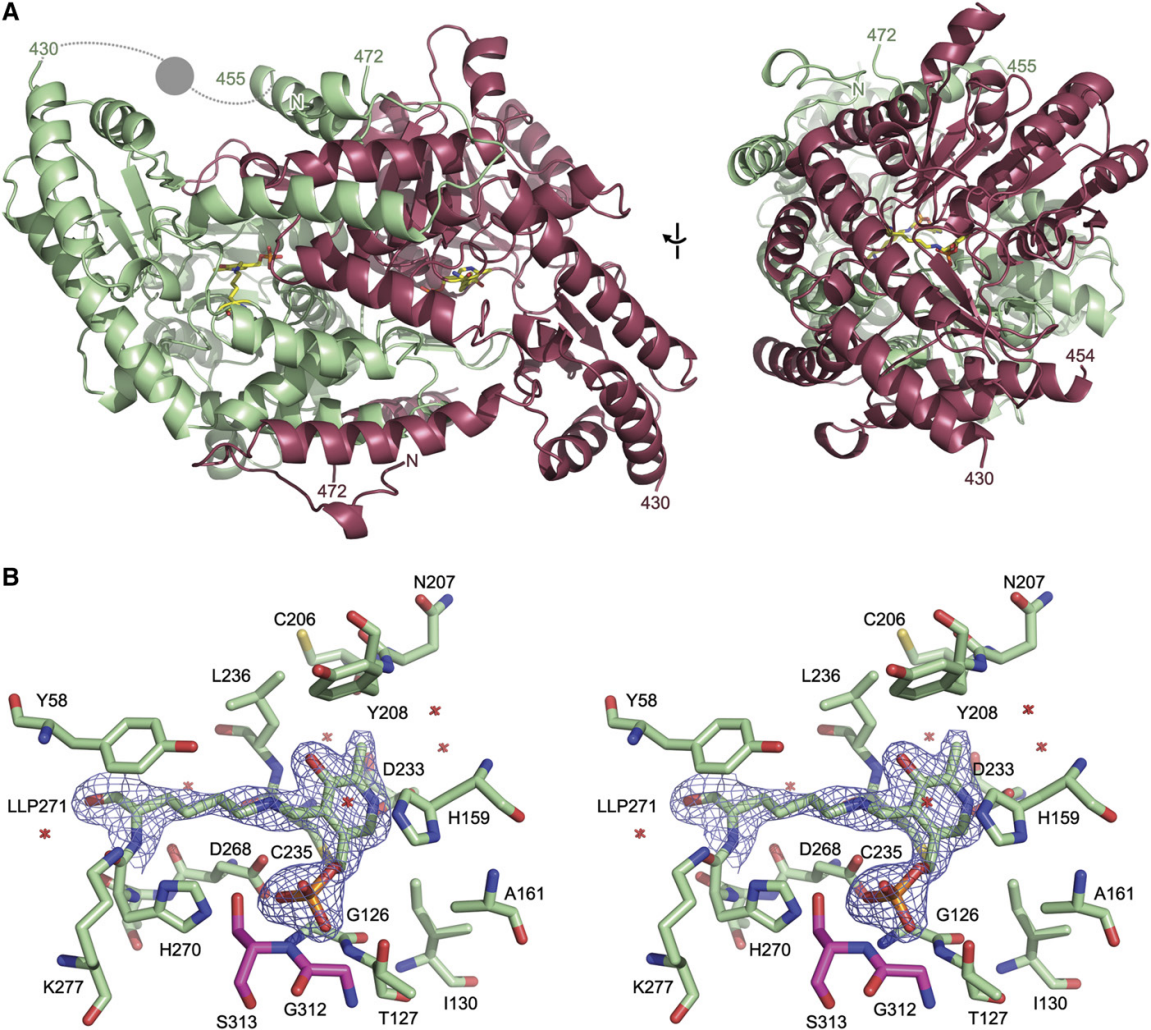
Structure of S1PL2021. A: Orthogonal views of the secondary structure of the S1PL2021 homodimer (PDB code, 5K1R). The two chains present in the asymmetric unit are shown as cartoon representations in green and red. The PLP cofactor bound as an internal aldimine to K271 is shown as sticks (LLP271). The region of the protein between residues 430 and 455 that was absent from the experimental electron density is modeled based on homologous structures; the dashed lines represent loops and circle represents an α helix. B: Stereo view of the PLP-binding site of S1PL2021. Residues within 4 Å of the PLP cofactor are shown as sticks. Water molecules in this site are shown as crosses. The experimental 2mFo-DFc electron density is shown contoured at 1.5 σ around the internal aldimine formed between the PLP and K271 (LLP271). The PLP binding site is at the dimer interface and residues from each monomer are colored green and red.

To further characterize the *B. pseudomallei* S1PL enzymes, we subjected S1PL2021 to crystallization trials using commercially available screens. Crystals with a monoclinic morphology were grown in 20% (v/v) PEG400 and 10% (v/v) 1-propanol. Diffraction data on cryocooled crystals were collected on a single crystal at Diamond Light Source (beamline I03) to a maximum resolution of 2.1 Å in space group P1 2_1_ 1. The structure of S1PL2021 was determined by molecular replacement using a monomer of the *S. thermophilum* S1PL structure as the template (PDB code, 3MAD). The final refined model contains a dimer of S1PL with the PLP cofactor present as an internal aldimine with residue K271 in each chain (**Fig. 5A**). There was no clear electron density present for residues before L10, between residues H430 and A455, and for the His6 tag after residue P472 at the C terminus; residues in these regions were omitted from the final model (**Table 2, Fig. 5A**).

**TABLE 2.** S1PL2021 X-ray crystallographic data collection and refinement statistics

Wavelength	0.976
Resolution range	43.27–2.104 (2.179–2.104)
Space group	P 1 2_1_ 1
Unit cell	59.6 126.776 59.725 90 97.508 90
Total reflections	280,513 (25,538)
Unique reflections	50,729 (4,971)
Multiplicity	5.5 (5.1)
Completeness (%)	1.00 (0.98)
Mean I/sigma (I)	8.57 (1.87)
Wilson B-factor	32.55
R-merge	0.1336 (0.6925)
R-meas	0.1475 (0.7717)
CC1/2	0.994 (0.671)
CC*	0.999 (0.896)
Reflections used in refinement	50,714 (4,970)
Reflections used for R-free	2,637 (273)
R-work	0.1688 (0.2445)
R-free	0.2268 (0.3101)
CC (work)	0.947 (0.834)
CC (free)	0.932 (0.743)
Number of nonhydrogen atoms	7,190
Macromolecules	6,890
Protein residues	879
RMS (bonds)	0.013
RMS (angles)	1.22
Ramachandran favored (%)	97
Ramachandran allowed (%)	2.9
Ramachandran outliers (%)	0
Rotamer outliers (%)	1.8
Clashscore	3.26
Average B-factor	39.12
Macromolecules	39.17
Solvent	37.96

Values in parentheses are from the last resolution shell.

The S1PL2021 structure conformed to the typical type I PLP-dependent fold seen in the group II decarboxylase subfamily (11, 44). The overall architecture of the protein closely matches homologous structures, with excellent overall alignment of S1PL2021 with published structures (supplemental Fig. S8A, supplemental Table S1). The structure has an N-terminal domain (residues 10-55), which is primarily involved in dimerization, a large cofactor-binding central domain (residues 56-320), a short C-terminal domain (residues 321-430), and a C-terminal extension (residues 431-471) (Fig. 5A). The latter domain is partially disordered, with the region between residues 431 and 455 not visible in the experimental electron density; this region sits close to the cofactor and substrate-binding region and may change conformation on ligand binding.

The cofactor-binding site at the dimer interface shows excellent electron density for the PLP cofactor, which was found to bind to K271 via a Schiff base as an internal aldimine (Fig. 5B). Coupled with the high conservation of active site residues with other members of the S1PL family across bacteria, yeast, and human (supplemental Fig. S8B, supplemental Table S2), our structural data confirm the assignment of S1PL2021 as a member of the S1PL (EC number 4.1.2.27) and, given the high sequence identity of S1PL2025 (86%), suggest that this is also a S1PL family enzyme. The cluster of residues involved in PLP binding are highly conserved across the members of the S1PL family, with these residues adopting the same orientation in published crystal structures (supplemental Fig. S8B). Analysis of the active site shows that the pyridine ring is held in place between C235 and H159 with π-stacking interactions to the latter residue; the nitrogen of the pyridine group forms a hydrogen bond to D233 (Fig. 5B). The PLP phosphate group forms hydrogen bonds with the sidechain of H270 and the backbone nitrogens of G126, T127, and S313 from the partner chain. The K271 chain is held in place through a hydrogen bond between D268 and the sidechain nitrogen that forms the Schiff base with PLP.

The dimer observed in the crystal structure of S1PL2021 is the native form of this protein (Fig. 5A), as the cofactor-binding site and ligand-binding funnel is formed between the two monomers (supplemental Fig. S9A, B). The dimerization interface buries nearly 50% of the total surface area of each monomer with a total buried area of 6,430 Å^2^; the interface is stabilized by 52 hydrogen bonds and 32 salt bridges. The observation that S1PL2021 is unable to bind PLP when expressed in the full-length form, as annotated in the genome, is explained by the fact that any significant extension of the N terminus of the structure from that seen in the crystal structure would potentially occlude the substrate binding funnel and PLP binding site (supplemental Fig. S9A). The substrate-binding funnel of S1PL2021 is formed between the two monomers, with one monomer providing a primarily positively charged surface and the other a negatively charged surface (supplemental Fig. S9B). The missing region of the protein that forms the C-terminal extension is likely to play a role in substrate recognition, given its position over the funnel and its inherent flexibility in this structure.

## DISCUSSION

SLs and ceramides have been shown to be important players in various eukaryotic cell functions. The amino alcohol core structure of all SLs is built from L-serine and a long chain fatty acid and further elaborated by a series of enzymes that increase their complexity by phosphorylation, oxidation, and acylation (1). Cells maintain a balance of SLs and ceramides via highly regulated synthetic and metabolic pathways whose control mechanisms are still not fully understood. Perturbation of this balance has now been linked to a number of diseases such as cancer, atherosclerosis, autoimmunity, and infection (5, 6). A key regulatory mechanism involves the breakdown of the potent signaling molecule, S1P, by S1PL and *S. cerevisiae* has been a useful model organism where the pathways for de novo biosynthesis, recycling, and degradation of SLs have been delineated (47). Indeed, the first S1PL gene (*dpl1*) was characterized in *S. cerevisiae* by Saba et al. (9) and they showed that a Dpl1 mutant strain was not only highly sensitive to exogenous sphingosine, but also accumulated intracellular S1P upon exposure to sphingosine. In contrast to higher order organisms, relatively few studies have been carried out to investigate the role of SL biosynthesis and metabolism in bacterial species. Of these, it is interesting to note that microbes from the human microbiome, *P. gingivalis* from the oral cavity and the gut commensal *B. fragilis*, make SLs with unusual structural features (14–16). Their roles in bacterial metabolism, as well as their interaction within the host environment, are currently under investigation (48).

*Burkholderia* are a group of interesting bacterial species that can cause life-threatening infections and have a reputation as being highly resistant to antibiotics (49–51) They appear to lack the ability to synthesize de novo SLs because analysis of their genomes suggests that the biosynthetic genes are absent. We were therefore interested to discover that *B. pseudomallei* K96243 encodes two highly homologous ORFs that display moderate (∼40%) sequence identity to the *S. thermophilum* S1PL query sequence (supplemental Figs. S1, S2). The two ORFs, BPSS2021 and BPSS2025 (annotated as decarboxylases in the genome), also display 86% sequence identity to each other. Because a more detailed sequence and structural analysis revealed that both ORFs contained conserved residues important for binding the PLP cofactor and enzyme catalysis, we hypothesized that these genes encode S1PLs. Therefore, we cloned, expressed, and purified recombinant forms of both S1PLs and UV-vis analysis showed that they bound the PLP cofactor (supplemental Fig. S3). Monitoring the changes in the UV-vis spectrum of PLP-dependent enzymes can be a useful tool to study how substrates, products, and inhibitors interact with these enzymes. The PLP binding region of holo-S1PL2021 showed changes upon addition of 100 μM S1P with an initial sharp rise in the 330 nm region, followed by a slower increase at this wavelength (Fig. 2A). At the same time, there was a decrease in the absorbance at 420 nm. Addition of the product PE to S1PL2021 led to the appearance of a peak at 320 nm and a decrease at 420 nm with a shift to 395 nm (Fig. 2B). We carried out the same substrate and product binding analyses with S1PL2025 (Fig. 2C, D) and the general trend was similar; this was unsurprising, as they are 86% identical. In contrast, in their studies with the *S. thermophilum* S1PL, Bourquin et al. (44) observed the transient appearance of two peaks (403 and 420 nm), which they tentatively assigned as the PLP-S1P and PLP-PE external aldimines in the putative S1PL reaction mechanism (intermediates 3 and 7 respectively in supplemental Fig. S4). These peaks appeared immediately upon S1P addition, and then reduced over 5 min until the spectrum resembled that of the starting PLP-bound holo-form of the enzyme. Moreover, they noted that the yeast S1PL UV-vis spectrum did not show any changes upon S1P addition and they concluded that this enzyme was inactive. Our MS and FALDH coupled assay (described below) prove that both *Burkholderia* S1PL2021 and S1PL2025 are active. Given that the S1PLs did not regenerate the holo-form spectrum, it suggests differences between the *Burkholderia* S1PLs and the *S. thermophilum* S1PL with which they share ∼40% sequence identity. Our results could indicate the slow release of products (PE or 2E-HEX) or the buildup of a hitherto uncharacterized intermediate.

The ability to measure S1PL activity using a convenient method would help to expand structural and mechanistic studies of the enzyme in all organisms. Since the first discovery of S1PL activity in mammalian cells by Stoffel, Lekim, and Sticht (52), who used a ^14^C-labeled substrate, a number of S1PL assays have been developed that use radioactive, fluorescent, and fluorogenic substrates and substrate analogs. One of the earliest methods used incorporation of ^3^H labels into the S1P substrate and TLC and liquid scintillation counting analysis of the products. This was applied to the study of the S1PL enzyme in rat liver and values of *K_M_* = 9 μM and *V*_max_ = 0.082 pmol/min/μg were determined (22). Bandhuvula, Fyrst, and Saba (23) synthesized a fluorescent NBD-labeled S1P homolog (S1P-C18NBD) that was used to analyze the human S1PL in HEK293 cells and gave values of *K_M_* = 14.6 μM and *V*_max_ = 3.4 × 10^−4^ pmol/min/μg. These authors then prepared a more stable fluorescent S1P-C14 BODIPY analog that was used to analyze the human enzyme again in HEK293 cells that gave values *K_M_* = 35 μM and *V*_max_ = 5.1 × 10^−4^ pmol/min/μg, in good agreement with the previous study (24). An alternative clever strategy retained the phosphorylated amino alcohol structure of the natural S1P substrate and combined it with an ether-linked coumarin-based reporter to generate a substrate that, upon S1PL-catalyzed cleavage, breaks down by β-elimination to a highly fluorescent umbelliferone product. Although highly sensitive, this nonnative substrate displays a high *K_M_* (152 μM) for the S1PL in mouse embryonic fibroblasts and the fluorescence was significantly reduced in the presence of Triton X-100 that is used to solubilize the S1P (25). MS-based techniques have also been developed that involve the derivitization of the 2E-HEX aldehyde product with reagents (usually hydrazides) that improve ionization efficiency. The first MS-based method was developed by Berdyshev et al. (26) to characterize the S1PL activity in mouse liver microsomal extracts (*K_M_* = 5.7 μM, *V*_max_ = 0.171 pmol/min/μg). A similar GC-electron ionization MS method used pentafluorobenzoyloxime derivitization of the 2E-HEX to analyze the S1PL in mouse embryonic fibroblasts (*K_M_* = 6 μM, *V*_max_ = 0.374 pmol/min/μg) (27). Novartis has recently identified the human S1PL as an attractive target for the development of therapeutics and developed a high throughput MS-based method to screen for novel inhibitors (11). By using nicotinylhydrazide (Isoniazid) modification of the 2E-HEX, they obtained the first data on purified recombinant human S1PL (*K_M_* = 5.2 ± 0.9 μM). Most recently, Suh et al. (53) developed a stable isotope dilution method using an internal D-labeled standard to also determine the values for recombinant human S1PL in good agreement with the value determined by Novartis (*K_M_* = 4.67 μM, *V*_max_ = 2.6 pmol/min/μg).

We decided to use an MS-based method to confirm that the recombinant *B. pseudomallei* S1PL2021 isoform catalyzed the degradation of S1P to 2E-HEX (Fig. 3). Because this end-point method involves a number of liquid handling steps (derivitization, organic extraction) and requires an internal standard, we sought to develop a convenient continuous spectroscopic assay that could be used for S1PL analysis. Our choice of coupling enzyme was informed by our knowledge of the SL degradative pathway (Fig. 1). In mammals, the 2E-HEX aldehyde product of the S1PL enzyme is oxidized to the acid by a FALDH (also known as ALDH3A2; UniProt code, P51648) in an NAD^+^-dependent manner that can be monitored at 340 nm. Human FALDH has been the subject of various studies because it has been linked to the SL-metabolic disease, Sjögren-Larsson syndrome (8). Previously, recombinant full-length human FALDH had been isolated by Lloyd et al. (46), who showed that extraction of the enzyme from *E. coli* required the use of the anionic detergent, N-lauroylsarcosine. This enzyme displays broad substrate specificity and can use saturated fatty aldehydes of varying chain lengths (from C5 to C18) with *K_M_* values in the low micromolar range (4–35 μM) and *k*_cat_ values of ∼1 s^−1^ that would deem it suitable as a coupling enzyme. We prepared recombinant N-terminally His-tagged human FALDH by relatively straightforward soluble expression in *E. coli*, once we had removed the C-terminal 36 residues that contained a predicted potential TM domain (supplemental Fig. S5). Of note, Lloyd et al. (46) did not prepare 2E-HEX, but did show that the FALDH used the commercially available unsaturated *cis*-11-hexadecenal as a substrate. We found that our truncated recombinant FALDH was not only active with the saturated C14 and C16 fatty aldehydes, but, more importantly, also used the synthetic 2E-HEX as a substrate and we determined kinetic values (*K_M_* = 19.4 ± 3.2 μM and *k*_cat_ = 0.22 ± 0.01 s^−1^) that suggested that it would make a suitable coupling partner for the determination of S1PL activity (Fig. 4A, B).

During our work, a detailed X-ray structure and mechanistic study of the human FALDH was published with a view to understanding the substrate specificity of the enzyme and the impact of mutations that cause Sjogren-Larsson syndrome (54). In this report, the FALDH construct contained an N-terminal streptavidin affinity tag and, in comparison to our truncation, only removed the C-terminal 22 amino acids, the minimum based on the proposed TM region. This construct was catalytically active with C8, C12, and C16 saturated aldehyde substrates in the presence of 0.1% Triton X-100, but they noted that removal of a 40 amino acid section resulted in a drastic reduction in activity with the longer acyl-chain length substrates. The X-ray structure of the FALDH (PDB code, 4QGK; 2.1 Å resolution) revealed that, as well as being involved in the TM interaction, the C-terminal domain also contained a gatekeeper helix that is proposed to be involved in substrate specificity (supplemental Fig. 5B). This structure allowed us to rationalize how our truncation of the C-terminal hydrophobic element increased the solubility of our FALDH construct while retaining enough of the helix to maintain good activity toward long acyl chain substrates, crucially including 2E-HEX (supplemental Fig. S5A, B). We combined our FALDH with the purified *Burkholderia* S1PL2021 and S1PL2025 enzymes in the presence of PLP, NAD^+^, and S1P solubilized in Triton X-100. We observed that the assay was linear over a practical substrate range (0–175 μM) and determined kinetic values for S1PL2021 (*K_M_* = 48.2 ± 9.4 μM and *k*_cat_ = 0.015 ± 0.003 s^−1^) and S1PL2025 (*K_M_* = 50.2 ± 8.3 μM and *k*_cat_ = 0.008 ± 0.0005 s^−1^) (Fig. 4C, D; Table 1). Because both isoforms display such high sequence identity, it is not surprising that they display similar *K_M_* values for S1P; however, we noted that the S1PL2025 was 50% slower than the S1PL2021 isoform. Although we used this assay to only study the *Burkholderia* S1PLs, we think that this convenient FALDH-coupled method will be of use to those working with S1PLs from other organisms (e.g., yeast and human). It will be interesting to determine whether it can also be applied not only to purified recombinant enzymes and HTP S1PL inhibitor screens, but also to whole cell lysates.

To explore the structures of these novel *Burkholderia* S1PLs, we used our optimized expression system for S1PL2021 to prepare crystals that diffracted to 2.1 Å. Using the *S. thermophilum* S1PL structure (PDB code, 3MAD) as a template for molecular replacement, the structure refined well and the enzyme displayed the canonical type I fold of the PLP superfamily of enzymes (Fig. 5A, supplemental Fig. 8A) (55). Furthermore, it belongs to the group II decarboxylase subfamily, which is typical of S1PLs from other species. The monomer has a three domain architecture: a short N-terminal domain, a large central domain that binds the well-resolved PLP cofactor bound as an internal aldimine/Schiff base to K271, and an extended C-terminal domain (Fig. 5A). As well as K271, comparison with other S1PLs identifies conserved residues involved in PLP binding: C235 and H159 bind to the pyridine ring, residues G126, T127, H270, and S313* (from the other subunit) coordinate the phosphate group, and D233 is involved in salt bridge formation of the pyridine nitrogen (Fig. 5B, supplemental Table S2). An overlay of all the well-refined structures of S1PLs in the PDB with PLP and/or Pi bound shows that the S1PL2021 active site architecture is well-conserved (supplemental Fig. S8B, supplemental Table S2). Given its role in anchoring the PLP cofactor, it is not surprising that mutation of the conserved K271 drastically reduces S1PL activity to 2% when compared with wild-type (Fig. 4E). Both S1PLs showed activity with S1P as a substrate, but we also wished to explore to determine whether the phosphate of S1P was required for activity. Because we had observed binding of D-*erythro* sphingosine to S1PL2021 (Fig. 2E) by UV-vis spectroscopy, it was interesting to find that this isoform did catalyze breakdown of this nonphosphorylated SL (∼8% activity compared with S1P, Fig. 4F). Thus, it appears that the *Burkholderia* S1PLs display some substrate promiscuity and may be able to break down a range of both phosphorylated and nonphosphorylated SLs. In the future, it will be interesting to carry out a comprehensive substrate analysis with these enzymes and explore their specificity with respect to acyl chain length, C1 head group, the oxidation state at C4-C5 (sphingosine/sphinganine), and the C2-C3 stereochemistry (*erythro*/*threo*).

Although the crystal structures of S1PLs from *S. thermophilum*, *S. cerevisiae*, humans, and *L. pneumophila* have been solved, the exact nature of substrate binding and enzyme catalysis are still unclear because the structure of a S1P-bound complex has not been determined (11, 18, 44). For example, in the putative mechanism, the active site base that deprotonates the S1P hydroxyl leading to the retro-aldol-like cleavage of the substrate has not been identified (supplemental Fig. S4). Bourquin et al. (44) described a *S. thermophilum* S1PL:PLP-PE product complex as well as a S1PL:PLP internal aldimine with a phosphate ion bound and, together these structures, hinted at the residues involved in binding the phosphate of S1P. Despite screening numerous conditions, we were unable to trap the wild-type S1PL2021:PLP-S1P external aldimine complex because we assume the enzyme catalyzes the rapid breakdown of the S1P. We recently used mutagenesis of an active site lysine that allowed us to capture the PLP-dependent enzyme, serine palmitoyl transferase, bound to the inhibitor, myriocin (56). We used a similar strategy to screen crystals of the S1PL2021 K271A mutant for S1P binding, but this has so far failed to yield a structure (data not shown). To date, the exact residues involved in binding the S1P acyl chain are still not known, but an overlay of all available S1PLs suggests the presence of a hydrophobic funnel on the surface that leads down into the active site (supplemental Fig. S9). It is interesting that one of the differences between the two enzymes is at position 401; this is a histidine in S1PL2021 and a glutamine in S1P2025. This change could contribute to the 50% difference in activity between the two isoforms. In the future, swapping the histidine at position 401 for glutamine (and vice versa) between the two forms, as well as mutating other potential residues, should identify those involved in substrate binding and catalysis. Our continuous S1P assay will be useful for screening the activity of these and any other mutants.

Because human S1PL has recently been identified as an attractive target for drug discovery, both Novartis and AbbVie have undertaken medicinal chemistry programs to identify potent inhibitors (11, 57). The determination of the structures of human S1PL and of the “humanized” *S. thermophilum* S1PL in complex with various inhibitors (PDB codes: 4Q6R, 5EUE, and 5EUD, respectively) have highlighted how these molecules block the substrate-binding funnel. It will also be interesting to test these and other S1PL inhibitors against various bacterial S1PLs with a view to screen them for antibacterial activity.

To our knowledge, this is the first time that two highly-related S1PLs have been identified in the same organism. At present, this phenomenon appears to be exclusive to species within the *Burkholderia* genus. The *Burkholderia* genus encompasses a remarkably diverse and versatile group of bacteria, including environmentally-beneficial species as well as pathogens of animals, humans, and plants. It is notable that our search of available genomes identified that *Burkholderia-*encoded S1PLs are restricted to *B. pseudomallei* and *B. thailandensis*, both of which are facultative intracellular species. This is consistent with the bacterial S1PLs contributing to pathogenesis during infection of host cells, as has recently been described for *Legionella*-encoded S1PL (18). The ability to invade, survive, and replicate within host cells is central to the pathogenesis of *B. pseudomallei* (58) and, while *B. thailandensis* is a highly attenuated relative to *B. pseudomallei*, it displays a similar intracellular phenotype (59) and, as such, is a valuable model organism for studying the intracellular lifestyle of *B. pseudomallei*. SLs have been shown to be key players in cell homeostasis during stress, such as that caused by bacterial infection (60). They mediate cross-talk between apoptosis and autophagy, the two major mechanisms of controlled cell death (61). Recently, studies on *L. pneumophila*, the causative agent of Legionnaires’ disease, have sought to understand the role that the *L. pneumophila* S1PL plays in its ability to survive intracellularly (18). It was found that *L. pneumophila* S1PL activity alone prevented an increase in sphingosine levels in infected host cells and this led to an inhibition of autophagy during macrophage infection. This work revealed the disruption of host SL biosynthesis as a novel mechanism used by intracellular pathogens to inhibit autophagy.

Because *Burkholderia* appear to not produce SLs such as S1P, the identity of the “true” intracellular substrate(s) for these enzymes is the subject of conjecture. We have shown that S1PL2021 can also degrade sphingosine as well as S1P, and it will be interesting to study a comprehensive range of potential substrates with each S1PL isoform to define their specificity. *Burkholderia* would most likely encounter high concentrations of S1P only during infection of a mammalian host and this would require the S1PLs to be secreted. In a recent complementary study, we have discovered that the two S1PL homologs present in *B. thailandensis* (BTH_II0309 and BTH_II0311; supplemental Fig. S1) are found in the extracellular supernatant in a pH-dependent manner (62). We are currently investigating whether both of these S1PLs form part of a higher order complex as pairs of homo- or heterodimers, but initial native gel electrophoresis suggests the presence of larger complexes. We also found that when expressed alone, BTH_II0309 and BTH_II0311 could each partially restore resistance to 10 μM sphingosine treatment of a yeast *Dpl1* mutant and full resistance was displayed by cells expressing both S1PLs. Our future work will determine whether the *B. pseudomallei* S1PL2021 and S1PL2025 can also complement the yeast Dpl1 mutant and are secreted in a similar way to the *B. thailandensis* isoforms. We studied the functional roles of the *Burkholderia* S1PLs and found that they were required for virulence in a Galleria wax moth larvae model and survival in murine macrophages (62). Of particular relevance to the current study, we discovered that a *B. pseudomallei* S1PL2025 mutant displayed a much reduced virulence phenotype (80% survival) compared with the wild-type organism (100% mortality) using a murine infection model in BALB/c mice. We are currently constructing the *B. pseudomallei* S1PL2021 knockout and a double mutant to study their virulence phenotypes. In summary, our work has characterized two S1PL homologs in *B. pseudomallei* and, when combined with recent studies on the emerging roles of bacterial S1PLs in pathogenesis, suggests that these enzymes merit further study.

## Supplementary Material

Supplemental Data
