# Supplementary material for: Characterization of homologous sphingosine-1-phosphate lyase isoforms in the bacterial pathogen Burkholderia pseudomallei
Source: J Lipid Res. 2016 Dec 29;58(1):137–50. doi: 10.1194/jlr.M071258 (PMC5234717; doi:10.1194/jlr.M071258)
Supplement: Supplemental Data [file supp_58_1_137__index.html]

Characterisation of homologous sphingosine 1-phosphate lyase (S1PL) isoforms in the bacterial pathogen Burkholderia pseudomallei. — Characterization of homologous sphingosine-1-phosphate lyase isoforms in the bacterial pathogen Burkholderia pseudomallei — Supplemental Data 

# Characterization of homologous sphingosine-1-phosphate lyase isoforms in the bacterial pathogen *Burkholderia pseudomallei*

## Supplemental Data

- Supplementary Tables and Figures (.pdf, 3.8 MB) - Tables, Figures
