# Supplementary material for: Characterization of homologous sphingosine-1-phosphate lyase isoforms in the bacterial pathogen Burkholderia pseudomallei
Source: J Lipid Res. 2016 Dec 29;58(1):137–50. doi: 10.1194/jlr.M071258 (PMC5234717; doi:10.1194/jlr.M071258)
Supplement: Supplemental Data [file 10.1194_M071258_jlr.M071258-1.pdf]

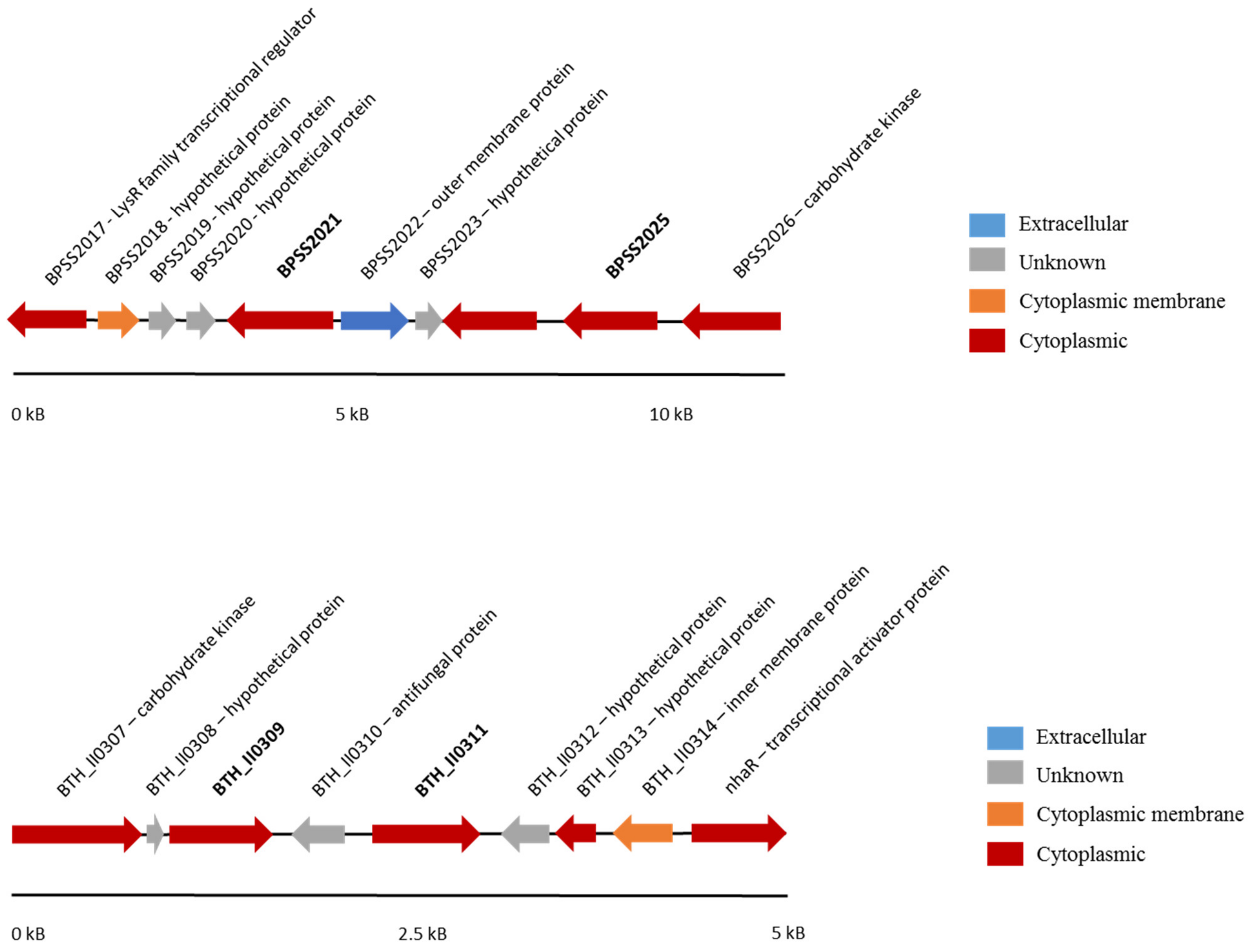

Supplementary figure S1. Putative S1PL coding region of *B. pseudomallei* K96243 genome. Top The genome of *B. pseudomallei* K96243 contains two open reading frames (ORFs), BPSS2021 and BPSS2025, whose gene products (named S1PL2021 and S1PL2025 respectively) contain 86% sequence identity with each other and ~44% sequence identity to the known S1PL from *S. thermophilum, St*S1PL (Uniprot code: Q67PY4). **Bottom** The genome of *B. thailandensis* E264 contains two ORFs, BTH_II0309 and BTH_II0311 (named S1PL0309 and S1PL0311 respectively), whose gene products contain 87% sequence identity with each other and contain ~44% sequence identity to *St*S1PL.

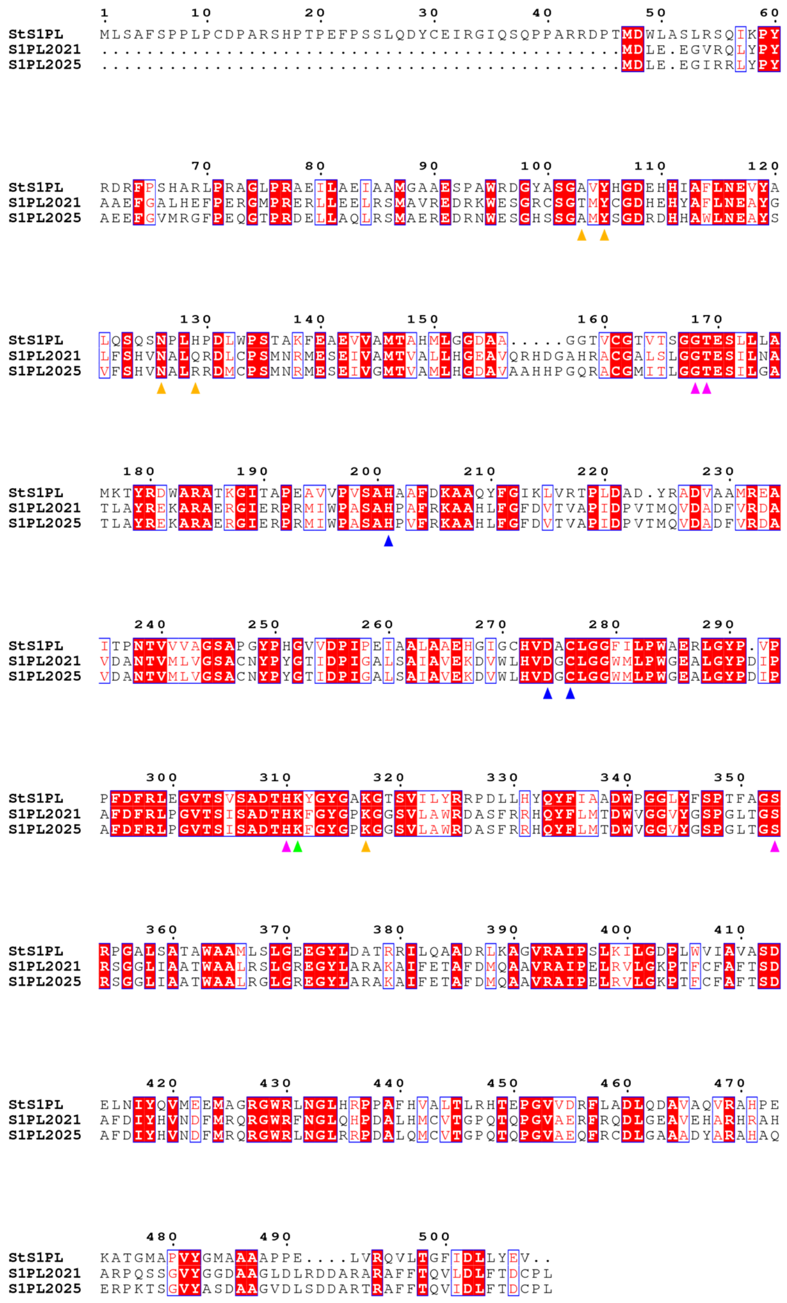

Supplementary figure S2. Protein sequence alignment between *St*S1PL and putative forms from *B. pseudomallei* K96243. The *B. pseudomallei* K96243 ORFs BPSS2021 (S1PL2021) and BPSS2025 (S1PL2025) from share ~86% sequence identity with each other and ~44% with *St*S1PL (Uniprot code: Q67PY4).Conserved residues include the catalytic lysine that binds pyridoxal 5'-phosphate (PLP) (green triangle), those that coordinate the PLP phosphate group, (pink triangle), others involved in stabilisation of the PLP ring (blue triangle) and important residues proposed to coordinate the phosphate moiety of the S1P substrate (orange triangle). The N-terminii have no homology with the pairwise alignment beginning at M47 (*St*S1PLnumbering).

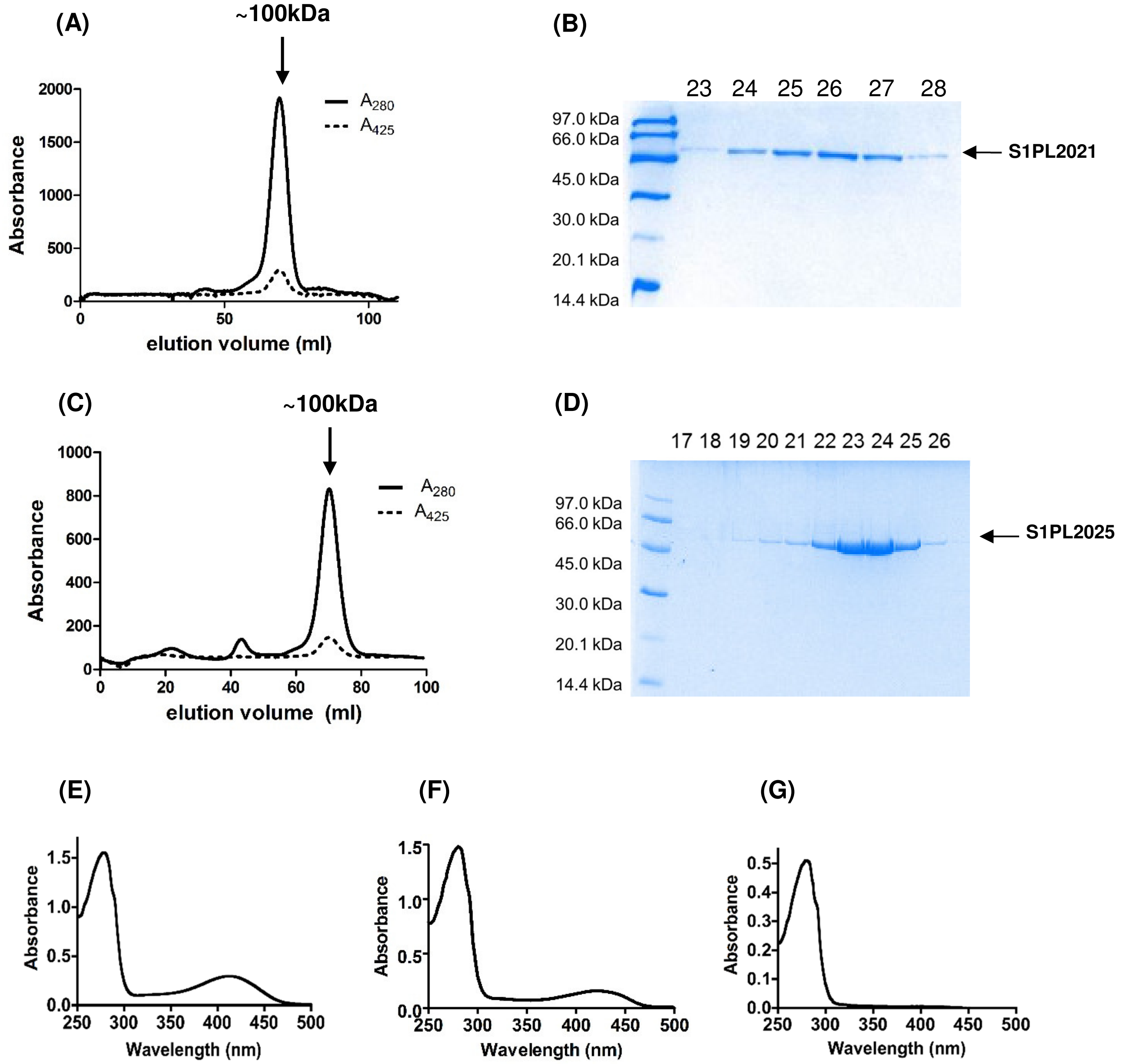

Supplementary figure S3. Purification of recombinant S1PL2021 and S2PL2025 and PLP cofactor binding UV-visible spectroscopy analysis. (**A and C**) Size exclusion chromatography (SEC on Superdex S200) analysis is consistent with S1PL2021 (**A**) and S1PL2025 (**C**) eluting as homo-dimers (~100 kDa) in solution. SDS-PAGE analysis of the fractions from the SEC analysis of S1PL2021 (**B**) and S1PL2025 (**D**) showing a monomer mass of ~50 kDa. UV-visible spectroscopy analysis shows that the purified truncated form of S1PL2021 (**E**) and S1PL2025 (**F**) display the characteristic absorbance maximum for binding the PLP cofactor (~420 nm). In contrast, purified S1PL2021 with an N-terminal extension (S1PL2021 N-EXT) does not bind PLP (**G**).

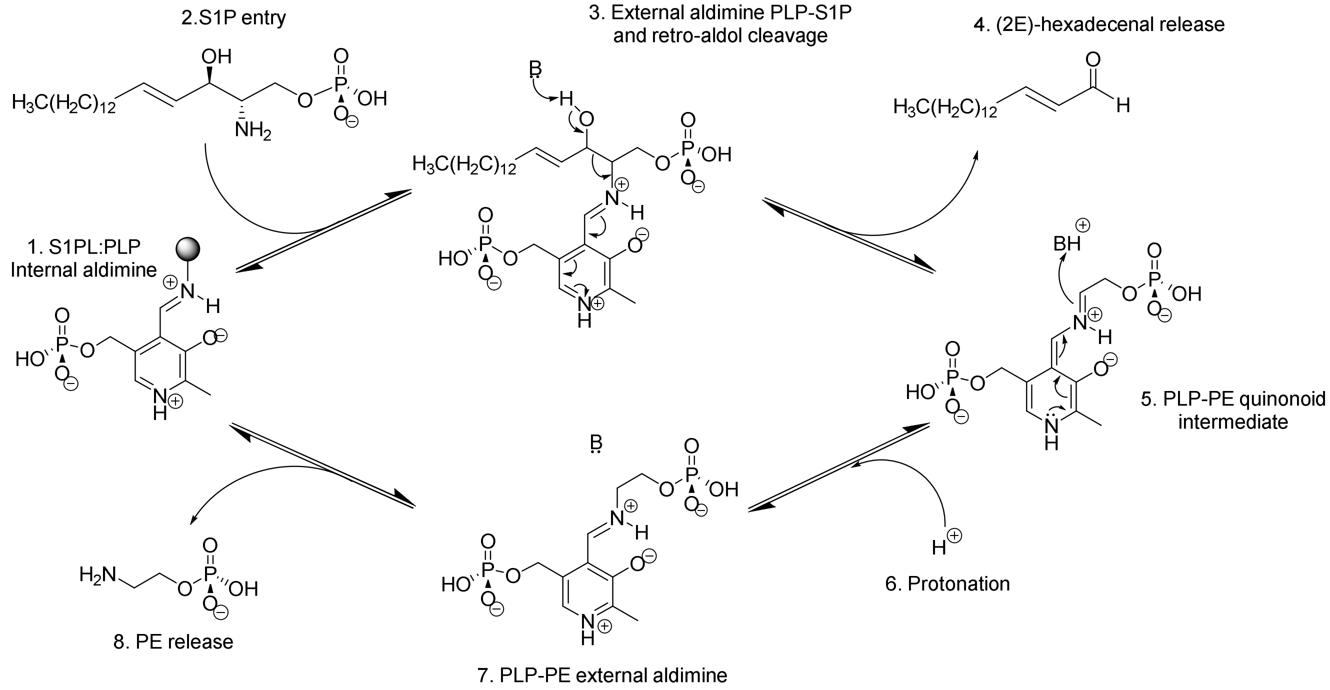

Supplementary figure S4. Proposed S1PL reaction mechanism. **(1)** In the holoform, PLP is bound to S1PL as an internal aldimine via a Schiff base linkage with the active site lysine. **(2)** Binding of the S1P substrate triggers transaldimination and formation of the S1PL:PLP-S1P external aldimine complex. **(3)** The PLP-S1P external aldimine intermediate is deprotonated by an unknown base at the C3 hydroxyl position in a retro-aldol cleavage mechanism. This releases the 2E-HEX product **(4)** and generates the product PLP-bound quinonoid intermediate **(5)**. Stereospecific protonation **(6)** of this intermediate gives the PLP-PE product external aldimine **(7)** followed by PE release **(8)** and reformation of the S1PL:PLP internal aldimine **(1)**.

(A)

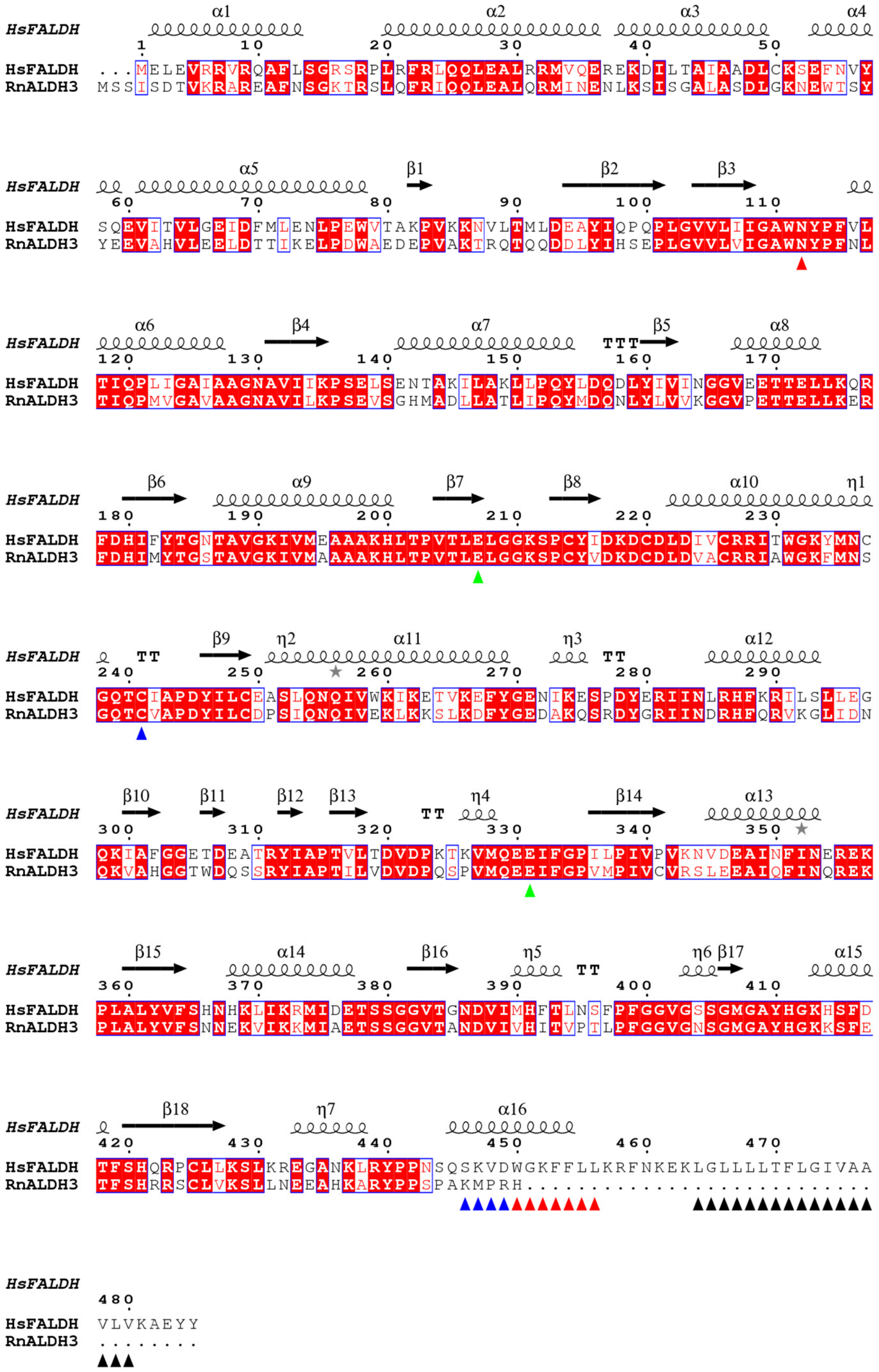
HsFALDH ...MELEVRRVRQAFLSGRSRPLRFRLQQLEALRRMVQEREKDILTAIAADLCKSEFNVY
RnALDH3 MSSISDTVKRAREAFNSGKTRSLQFRIQQLEALQRMINENLKSISGALASDLGKNEWTSY
HsFALDH SQEVITVLGEIDFMLENLPEWVTAKPVKKNVLTMLDEAYIQPQPLGVVLIIGAWNYPFVL
RnALDH3 YEEVAHVLEELDTTIKELPDWAEDEPVAKTRQTQQDDLYIHSEPLGVVLVIGAWNYPFNL
HsFALDH TIQPLIGAIAAGNAVIIKPSELSENTAKILAKLLPQYLDQDLYIVINGGVEETTELLKQR
RnALDH3 TIQPMVGAVAAGNAVILKPSEVSGHMADLLATLIPQYMDQNLYLVVKGGVPETTELLKER
HsFALDH FDHIFYTGNTAVGKIVMEAAAKHLTPVTLELGGKSPCYIDKDCDLDIVCRRITWGKYMNC
RnALDH3 FDHIMYTGSTAVGKIVMAAAAKHLTPVTLELGGKSPCYVDKDCDLDVACRRIAWGKFMNS
HsFALDH GQTCIAPDYILCEASLQNQIVWKIKETVKEFYGENIKESPDYERIINLRHFKRILSLLEG
RnALDH3 GQTCVAPDYILCDPSIQNQIVEKLKKSLKDFYGEDAKQSRDYGRIINDRHFQRVKGLIDN
HsFALDH QKIAFGGETDEATRYIAPTVLTDVDPKTKVMQEEIFGPILPIVPVKNVDEAINFINEREK
RnALDH3 QKVAHGGTWDQSSRYIAPTILVDVDPQSPVMQEEIFGPVMPIVCVRSLEEAIQFINQREK
HsFALDH PLALYVFSHNHKLIKRMIDETSSGGVTGNDVIMHFTLNSFPFGGVGSSGMGAYHGKHSFD
RnALDH3 PLALYVFSNNEKVIKKMIAETSSGGVTANDVIVHITVPTLPFGGVGNSGMGAYHGKKSFE
HsFALDH TFSHQRPCLLKSLKREGANKLRYPPNSQSKVDWGKFFLLKRFNKEKLGLLLLTFLGIVAA
RnALDH3 TFSHRRSCLVKSLLNEEAHKARYPPSPAKMPRH
HsFALDH VLVKAEYY

(B)

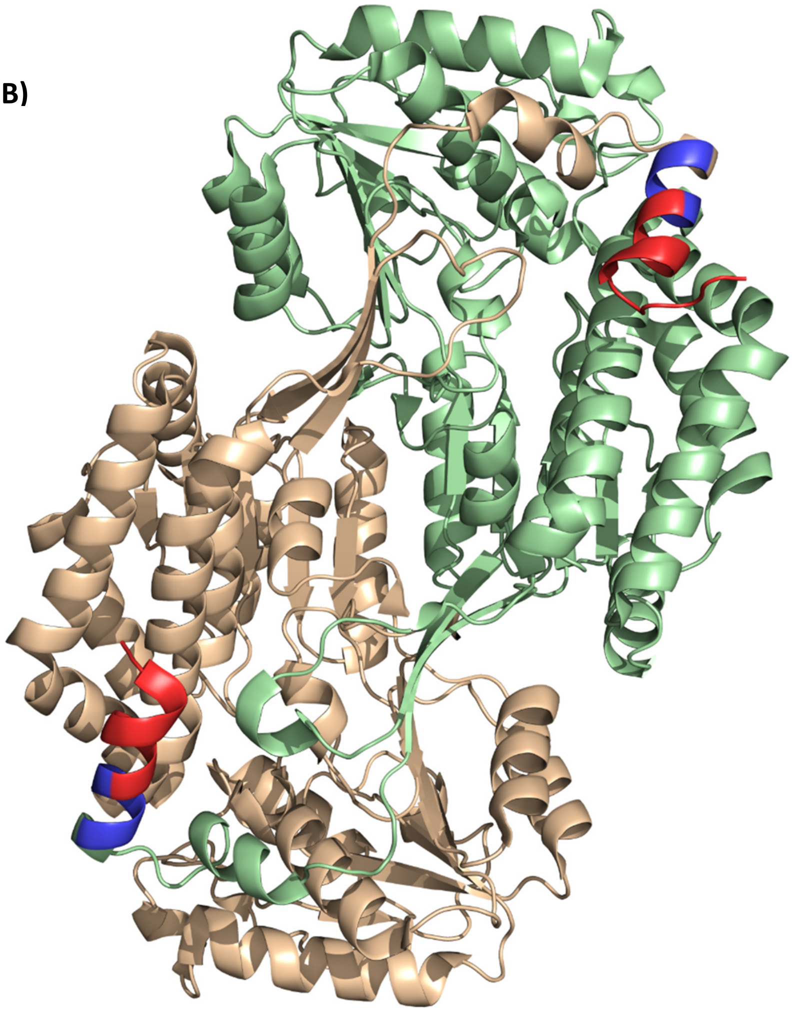

Supplementary figure S5. Sequence and structural alignment between *Rattus norvegicus* ALDH3 and human FALDH. (A) Preparation of a structural alignment using ESPript between human FALDH (Uniprot: P51648, PDB code: 4QGK) and rat ALDH3 (Uniprot: P11883) highlighted the extended C-terminus of the human protein. Key active site residues include the catalytic Cys241 (blue triangle) activated by either Glu207 or Glu331 (green triangle) and the aldehyde headgroup orienting Asn112 (red triangle). The transmembrane associated region is underlined (black triangle) while Keller's reported "gatekeeper helix" is underlined (red/blue triangle) with blue alone showing the truncation point of the recombinant FALDH used in this study. **(B)** Crystal structure of the human FALDH (PDB: 4QGK) highlighting the gatekeeper helix (red and blue). The recombinant FALDH we prepared lacks the region coloured red.

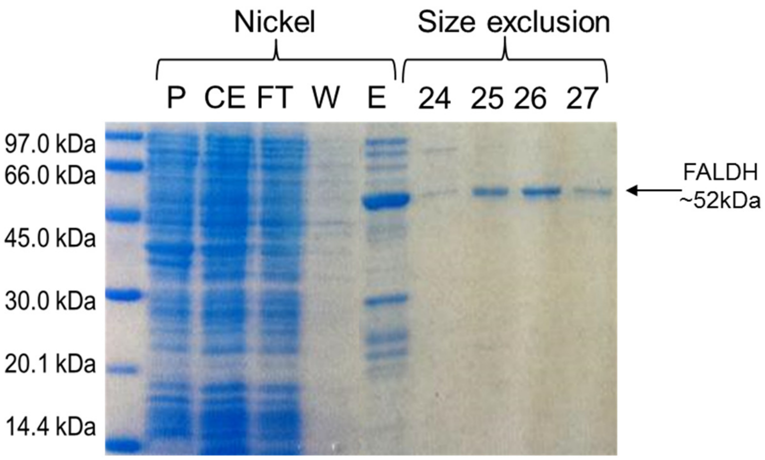

Supplementary figure S6. **Purification of truncated form of FALDH**. SDS-PAGE analysis of nickel affinity and SEC purification fractions showed the presence an over expressed band at ~52kDa corresponding to the FALDH protein construct used in this study. (P=pellet, CE=cell free extract, FT=flow through, W=wash, E=elution).

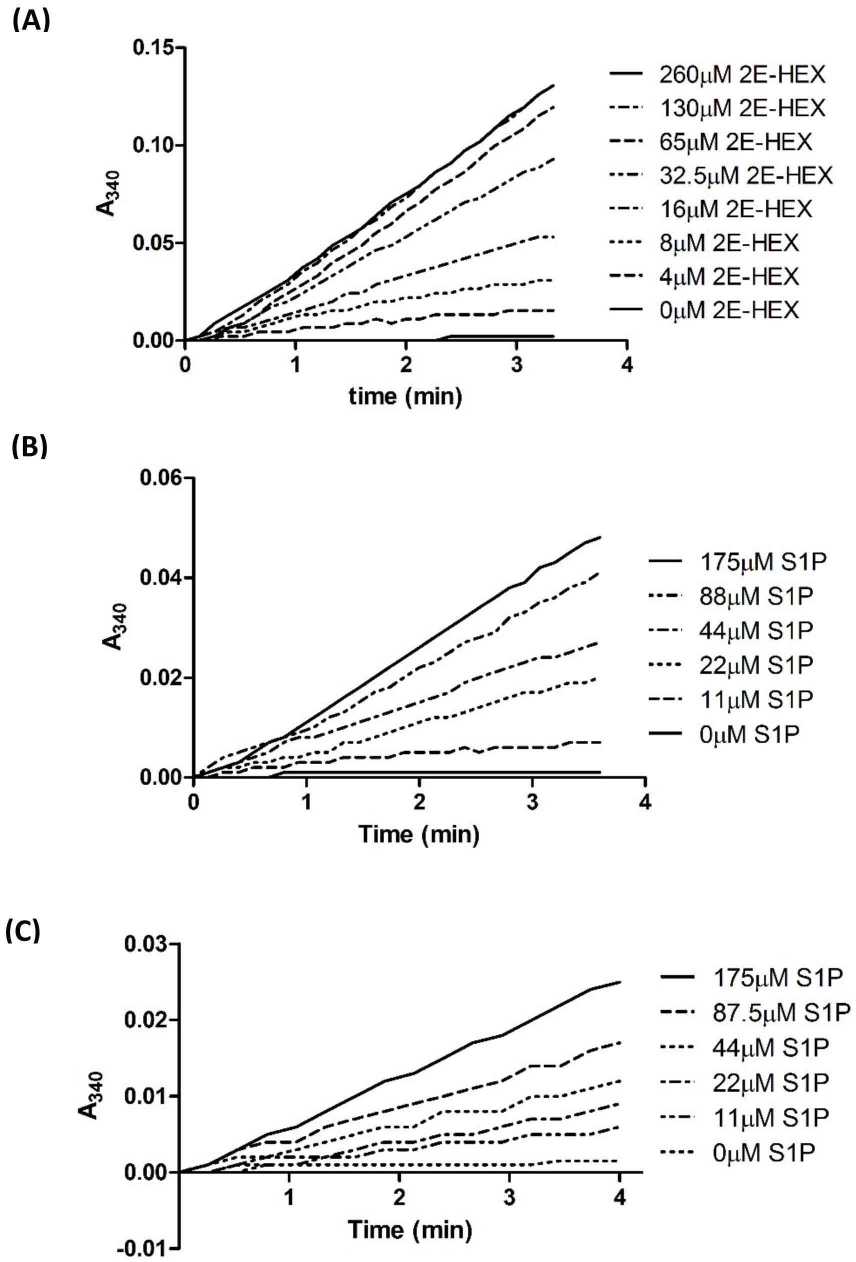

Supplementary figure S7. Monitoring absorbance changes at 340 nm to assay FALDH alone and in the S1PL/FALDH coupled assay. (**A**) Monitoring FALDH activity (0.5 μM) at 340 nm (NAD^+ to NADH conversion) shows the assay is linear between 4.0-260 μM 2E-HEX. **(B)** Activity of combined S1PL2021/FALDH enzymes shows the coupled assay is linear between 0-175 μM S1P and sensitive down to 10 μM. **(C)** Activity of combined S1PL2025/FALDH enzymes shows that the coupled assay is linear between 0-175 μM S1P and sensitive down to 10μM.

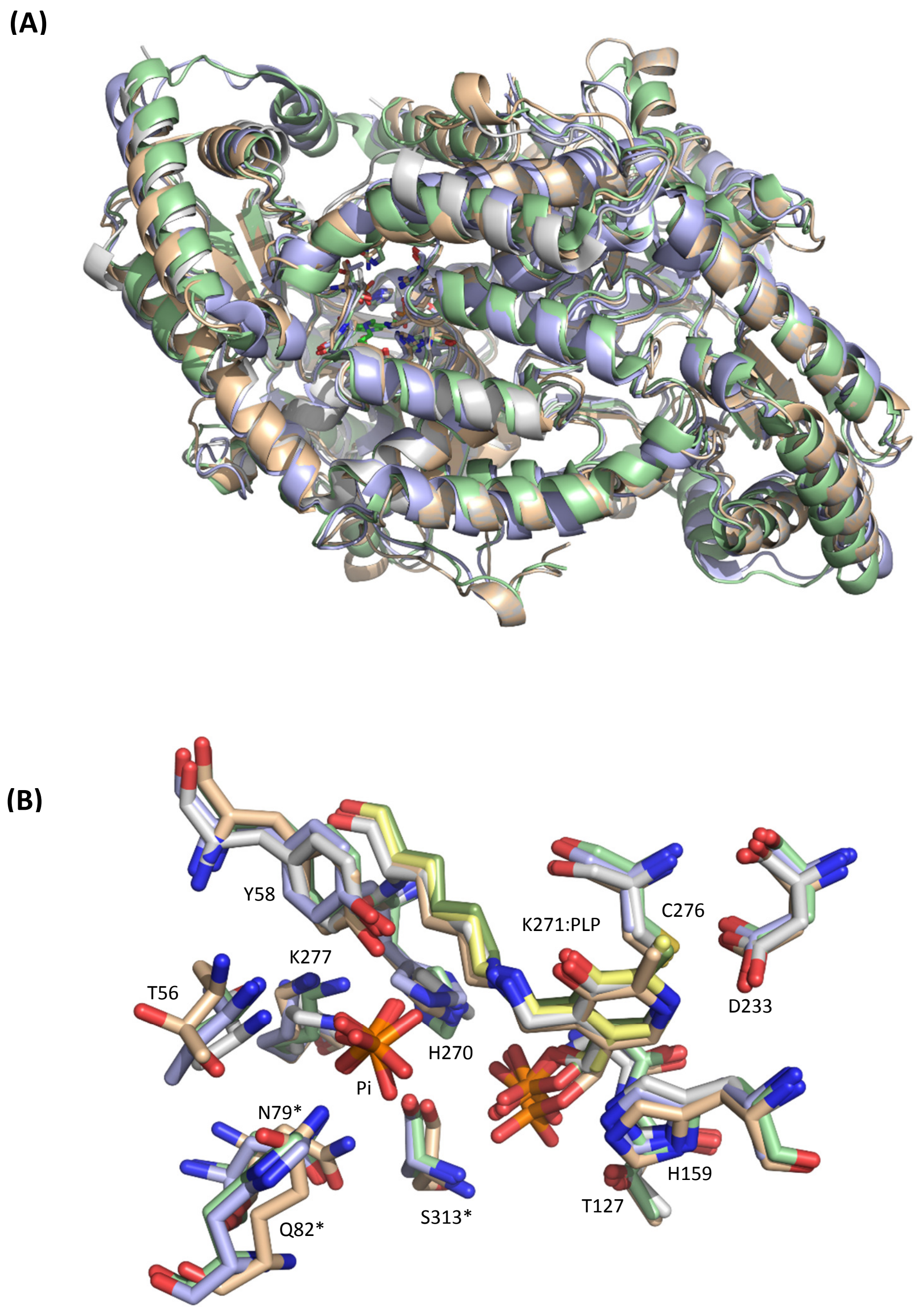

Supplementary figure S8. Structural alignment of S1PL homologues. (**A**) Cartoon view of aligned S1PL homologues whose x-ray structures have been determined. We used five well-refined structures of four enzymes; *Bp*S1PL2021 (PDB: 5K1R, this study, wheat colour); *St*S1PL (PDB: 5EUD, green, PDB: 3MAD, yellow); *Hs*S1PL (PDB: 4Q6R, blue) *Sc*S1PL (PDB: 3MC6, grey). The RMSDs and alignments are given in supplementary table S1. (**B**) Overlay of the S1PL active sites shows that the orientation and the residues involved in coordinating the PLP cofactor (T127, H159, K271, C276, D233, S1PL2021 numbering) are highly conserved. Those residues predicted to coordinate the S1P phosphate group, based on the *Sc*S1PL PLP:PE external aldimine

(PDB: 3MAU) and the free phosphate (Pi) observed in the S1PL structures are shown (T56, K277, Y58, S313, N79*, Q82*). The asterisk denotes a residue from other subunit within the homodimer. These are summarised in supplementary table S2.

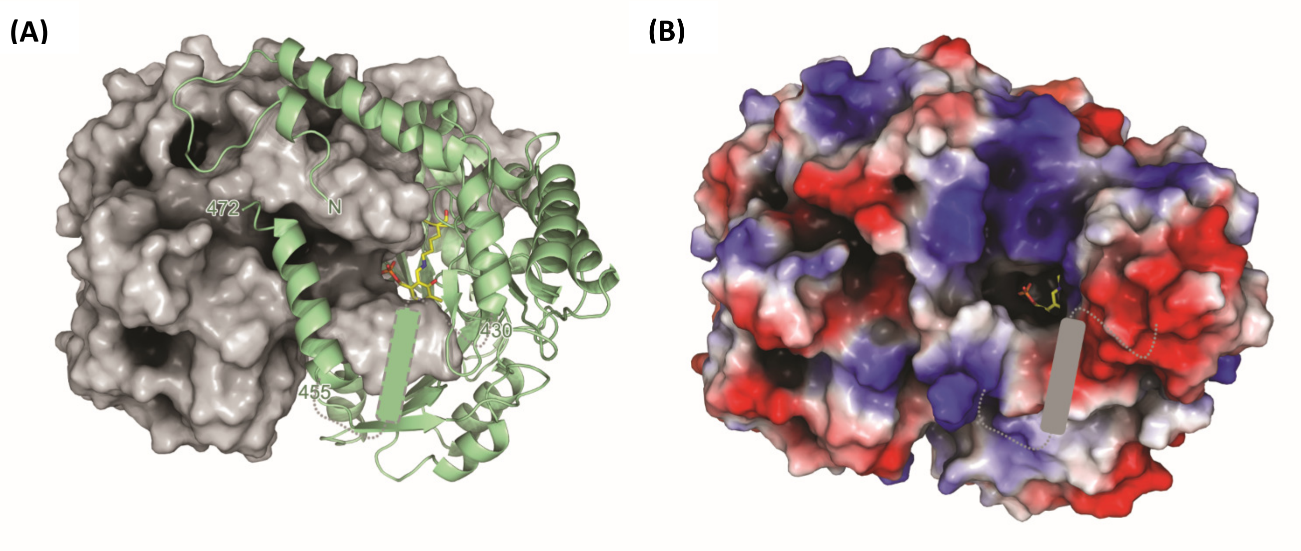

Supplementary figure S9. Surface views of S1PL2021. **(A)**View of S1PL2021 showing the surface of one monomer and the other as a cartoon with the PLP cofactor bound to K271 shown as sticks. The region of the protein between residues 430 and 455 that was absent from the experimental electron density is modelled based on homologous structures; the dashed lines represent loops and rounded rectangle represents an alpha helix. **(B)** Surface view of the dimer of S1PL2021 showing the electrostatic charge distribution. Blue indicates areas of positive charge, red negative, and grey neutral. The K271:PLP internal aldimine sits at the bottom of a cleft formed between the two monomers, modelled alpha helix in grey.

Structure	PDB ID	$RMSD_{C\alpha}$ (Å)	Number of residues aligned	Sequence identity (%)	Reference
**S1PL2021 (A vs B)**	5K1R	0.27	439	99.7	This study
***St*S1PL**	5EUD	1.04	426	46.0	59
***St*S1PL**	3MAD	0.94	424	46.4	44
***Hs*S1PL**	4Q6R	1.17	407	40.29	11
***Sc*DPL1**	3MC6	1.38	402	37.1	44
***Lp*S1PL**	4W8I	1.69	314	35 %	57

Supplementary table S1. A table listing comparison statistics (RMSDs, % sequence identity) of the S1PLs structures that have been deposited in the PDB.

(Proposed) Role	Organism					
	S. thermophilum (StSPL)	*S. cerevisiae* (Dpl1p)	*H. sapiens* (HsSPL)	*L. pneumophila* (LpSPL)	*B. pseudomallei (S1PL2021)*	*B. pseudomallei* (S1PL2025)
PLP Phosphate Binding Cup	G168	G235	G210	G220	G126	G126
	T169	T236	T211	T221	T127	T127
	H310	H379	H352	H359	H270	H270
	S353*	S422*	S395*	S402*	S313*	S313*
PLP Sandwich	C276	C344	C317	C328	C235	C235
	H201	H268	H242	H252	H159	H159
Substrate Phosphate Binding	Y105	Y174	Y150	Y160	Y58	Y58
	H129*	H198*	H174*	H185*	Q82*	R82*
	A103	A172	T148	A157	T56	A56
	N126*	N195*	N171*	N182*	N79*	N79*
	K317	K386	K359	K366	K277	K277
PLP Binding	K311	K380	K353	K360	K271	K271
PLP aspartate	D274	D342	D315	D326	D233	D233

Supplementary table S2. A table highlighting the active site residues conserved across various S1PLs.
